# Photomemristive sensing *via* charge storage in 2D carbon nitrides†

**DOI:** 10.1039/d2mh00069e

**Published:** 2022-04-26

**Authors:** Andreas Gouder, Alberto Jiménez-Solano, Nella M. Vargas-Barbosa, Filip Podjaski, Bettina V. Lotsch

**Affiliations:** Department Nanochemistry, Max Planck Institute for Solid State Research Heisenbergstr. 1 70569 Stuttgart Germany f.podjaski@fkf.mpg.de b.lotsch@fkf.mpg.de; Department Chemistry, Ludwig-Maximilians-University Butenandtstr. 5-13 81377 Munich Germany; Institute for Energy and Climate Research (IEK-12), Helmholtz Institute Münster, Forschungszentrum Jülich Corrensstr. 46 48148 Münster Germany

## Abstract

Photomemristive sensors have the potential to innovate current photo-electrochemical sensors by incorporating new sensing capabilities including non-invasive, wireless and time-delayed (memory) readout. Here we report the charge storing 2D carbon nitride potassium poly(heptazine imide), K-PHI, as a direct photomemristive sensing platform by capitalizing on K-PHI's visible light bandgap, large oxidation potential, and intrinsic optoionic charge storage properties. Utilizing the light-induced charge storage function of K-PHI nanosheets, we demonstrate memory sensing *via* charge accumulation and present potentiometric, impedimetric and coulometric readouts to write/erase this information from the material, with no additional reagents required. Additionally, wireless colorimetric and fluorometric detection of the charging state of K-PHI nanoparticles is demonstrated, enabling the material's use as particle-based autonomous sensing probe *in situ*. The various readout options of K-PHI's response enable us to adapt the sensitivities and dynamic ranges without modifying the sensing platform, which is demonstrated using glucose as a model analyte over a wide range of concentrations (50 μM to 50 mM). Since K-PHI is earth abundant, biocompatible, chemically robust and responsive to visible light, we anticipate that the photomemristive sensing platform presented herein opens up memristive and neuromorphic functions.

## Abstract 2

We present a photomemristive sensing concept based on light-induced charge storage in the 2D carbon nitride K-PHI. Our design enables memory sensing with tuneable dynamic concentration ranges, combined with diverse operation and readout modes.

## Graphical abstract

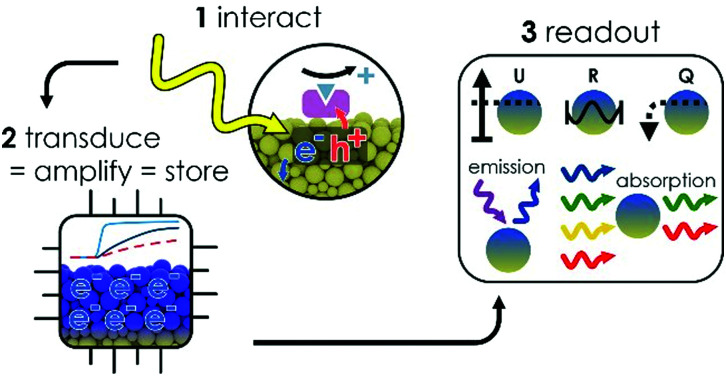

**New concepts**

We demonstrate a multimodal photomemristive sensing concept that bridges the field of (photo)electrochemical (PEC) sensing and memristive organic electronics. The carbon nitride poly(heptazine imide) (K-PHI) is capable of “traditional” PEC sensing of a wide range of organic analytes and at the same time shows a characteristic bifunctionality of light absorption coupled to photocatalytic reactivity, and charge storage. Using these unique “optoionic” properties, the sensing information (*i.e.* the stored charge) can be written onto the sensor and read out based on a number of physical quantities such as photovoltage or color change. This concept allows us to access new functionalities for sensors: memory of the analyte concentration information, tuneable sensitivities and dynamic sensing ranges, as well as a diverse array of readout methods. Due to the facile sensor geometry – either as films or wireless particles – we circumvent conceptual and technical challenges of existing PEC- or memristive sensing concepts. This work underlines how functionalities from different fields (batteries, photocatalysts, memristors, sensors) can be merged to produce information storing devices with novel functionalities. While this concept accelerates the rapidly emerging research field of memristive sensors into a new direction, it also presents a toolkit to facilitate automated electronic signal processing.

## 1. Introduction

The trend towards digitization and automation necessitates novel sensing and information storage concepts. Sensors are of critical importance for medical applications, in smart healthcare systems and environmental monitoring.^1–3^ Emerging neuromorphic sensing and monitoring concepts utilize memristive effects to enable exciting features such as memory sensing and low power data processing.^4–7^

The traditional path to application of memristive devices are non-volatile memory applications with many distinctly addressable states.^4^ Artificial synapses are a class of bio-memristive devices which are cheap, offer biocompatibility and are investigated to develop synaptic electronics such as neural networks for neuromorphic computing.^4,8–11^ However, memristors have further potential. Coupling photovoltaics with memristors or transistors can drastically reduce computational energy consumption with photonic- or photomemristive devices, which have been dubbed as “solar brains”.^12^ The memristive sensing approach presented in this work relies on such a photomemristive behaviour. Most current memristive sensors are comprised of classical inorganic, memristive materials (*e.g.* Si-nanowires) which are functionalized with a receptor and produce a voltage gap in their memristive behaviour that can be linked to the analyte concentration.^6^ However, those two-terminal memristor devices suffer from complex device geometries, as they do not have a straightforward pathway for biomolecules to approach the device.^13^ To address this challenge, complex surface patterning^14^ or nanowire^15^ designs are necessary. While most memristive sensors so far have focused on the detection of complex biomolecules such as DNA aptamers,^16^ cancer markers^17^ or the ebola and dengue virus,^18,19^ sensing of easier biomolecules such as glucose was demonstrated but with a lack in both sensitivity (10–40 mM) and selectivity.^20^

Organic memristive devices have been designed with several different voltage thresholds or volatile switching mechanisms.^4,5,21–23^ The common denominator is the writing and reading of different conductance states, which can be utilized for information storage. Redox-based switching uses electrochemical redox reactions of polymers (*e.g.* PEDOT:PSS)^4,23^ accompanied by ion diffusion, which modifies the conductance of the material.

Carbon nitrides are an interesting class of organic materials with potential applications in biosensing and memristive devices. They are layered molecular materials with one- (1D) or two-dimensional (2D) triazine- or heptazine backbones and a visible-light bandgap. Carbon nitrides have lately attracted interest in various research areas including photocatalysis,^24–27^ electrochemical and solar energy storage,^24,28,29^ molecular machines,^30^ environmental remediation,^31^ and non-enzymatic or ‘nanozyme’ sensing of *e.g.* glucose.^3,32–35^ Carbon nitride based sensors typically require heterostructures, mediators and/or redox indicator molecules. Potassium polyheptazine imide (K-PHI), a recently discovered 2D carbon nitride,^26,36,37^ has remarkable optoelectronic and optoionic properties^38^ that are derived from its dual functionality of light harvesting and charge storage.^24,28,39,40^ This versatile toolkit has led to the design of novel responsive and/or charge storing concepts, such as “dark photocatalysis”^23^ and a solar battery anode.^28^ K-PHI's large bandgap of ∼2.7 eV (see Fig. S1, ESI†) corresponding to an absorption edge in the blue region of the visible spectrum, together with the material's suitably positioned band edges (+2.2 and −0.5 V *vs.* NHE, for the valence band maximum and conduction band minimum, respectively), provides enough thermodynamic driving force for the oxidation and reduction of various chemical species,^41^ while being chemically robust.

**Fig. 1:**
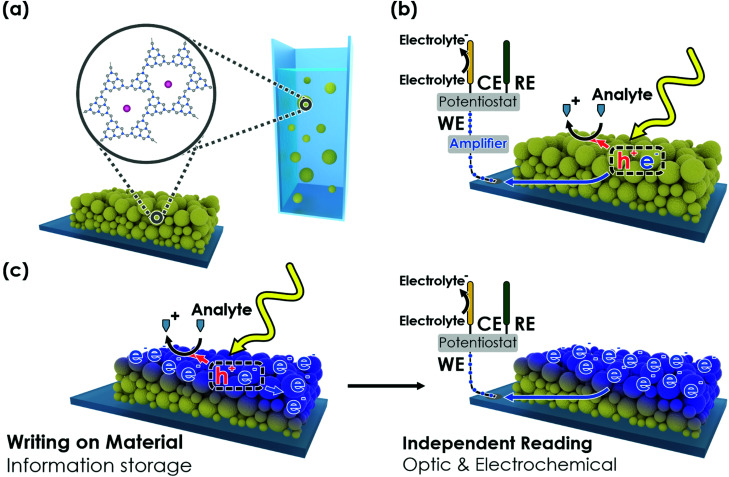
Concept of an oxidative (memristive) photo-electrochemical sensor. (a) The carbon nitride K-PHI can be deployed as nanosheets in a thin film on a conductive substrate or as nanoparticles in an aqueous suspension for wireless sensing. (b) Components of carbon nitride-based direct photo-electrochemical (PEC) sensors. Electron–hole pairs are generated upon illumination. The holes are extracted *via* oxidation of the analyte (*i.e.* K-PHI acts as a receptor). The electrons generate a signal proportional to the analyte concentration (*i.e.* K-PHI acts as a transducer). (c) In a photomemristive sensor, instead of generating a photocurrent directly, the electrons charge the sensor (writing) and thus, change the sensor's material properties as a function of the analyte concentration. This change is integrative due to accumulation of charge and modifies the material properties non-linearly (*i.e.* K-PHI acts as memristive amplifier). Subsequently, the material properties can be accessed *via* electrochemical or optical techniques (readout).

Herein, we exploit the abovementioned property modifications of K-PHI upon photocharging (Fig. 1(a)) and propose a refined photomemristive sensor beyond the commonly used two-terminal design. We show that K-PHI simultaneously acts as a receptor unit for glucose as a model analyte and a transducer, which is coupled to a memristive amplifier, thereby combining all sensing components in the same material. This compact design is complemented by various readout methods, which are based on different physical quantities that encode K-PHI's charging state. Our concept of sensing *via* charge storage comes with a novel set of combined features, such as an intrinsic memory function with writing and erasing procedures, tuneable dynamic concentration ranges and adaptive sensitivities – properties which we explore, demonstrate and explain below. The manuscript is structured as follows: we first briefly show the direct photo-electrochemical (PEC) sensing ability of K-PHI (Fig. 1(b)) with different analytes to explain the interaction of the sensor with the analyte. This allows us to demonstrate in the following the K-PHI memsensing concept (Fig. 1(c)) *via* potentiometric, impedimetric and coulometric techniques that focus on the modified electronic material properties. Finally, we present a wireless, memristive sensing mode using optical readouts (colorimetric and fluorometric). Thus, we connect the field of memristive information storage to the well-established electrochemical sensor design by moving away from the more complex “classical” memristor design to a direct PEC sensor with built-in memristive functionalities. This is only possible due to the combination of a special set of photo-electrochemical properties of the carbon nitride K-PHI, namely light absorption, charge storage and a large oxidative driving force.

## 2. Results and discussion

### 2.1. PEC sensing interaction on K-PHI

The concept of photo-electrochemical (PEC) biosensors is briefly summarized in Fig. 1(b).^44^ To describe the direct memristive sensing mode discussed here, we start by summarizing the capability of K-PHI nanosheet films (see Fig. 1(a) and Fig. S2, S3 for details, ESI†) to perform direct PEC amperometric sensing, *i.e.*, to simultaneously act as receptor and transducer while being illuminated in aqueous conditions (Fig. 1(b)). We use glucose as model analyte (see Section S2, ESI†), relating the analyte concentration to the photocurrent. Three linear concentration ranges are observed (0–1 mM, 1–10 mM, and 10–50 mM) and a limit of detection (LOD) of 11.4 μM (0.21 mg dL^−1^) can be determined from the lower concentration range.^45^ These observations are comparable to reported linear ranges and LOD's for metal-free amperometric carbon nitride glucose sensors (LOD of 11 μM and a linear range from 1 to 12 mM),^34^ which additionally require the mediator H_2_O_2_ in convolution with the enzyme glucose oxidase (GOx) as the receptor. To confirm the broad applicability and generality of K-PHI as a PEC sensor, we have also tested other sugars, alcohols, the physiologically relevant molecules uric acid and ascorbic acid, as well as typical electron donor molecules, which are widely used as sacrificial agents in photocatalytic applications (Fig. S4(d), ESI†). All these analytes show a strong photocurrent response and hence, can be detected with K-PHI sensors. At a concentration of 5 mM, sugars produce photocurrents between ∼0.6 and 1 μA cm^−2^ and the typical SED triethanolamine (TEoA) and 4-methylbenzyl alcohol (4-MBA) show photocurrents of 7.5 and 23 μA cm^−2^, respectively. The physiologically relevant molecules ascorbic acid (AA) and uric acid (UA) produce a photocurrent response of 0.38 and 0.35 μA cm^−2^, respectively, at a concentration of 0.1 mM, suggesting a viable monitoring strategy for these analytes as well. However, the strong oxidative driving force, which enables considerable photocurrents with many different species, makes it difficult to differentiate between organic substances in mixtures. Functionalization protocols for carbon nitrides developed by us^46^ and others^47^ are expected to further enhance the selectivity of K-PHI towards these analytes.

### 2.2. Photo-memristive sensing with K-PHI films

**Fig. 2:**
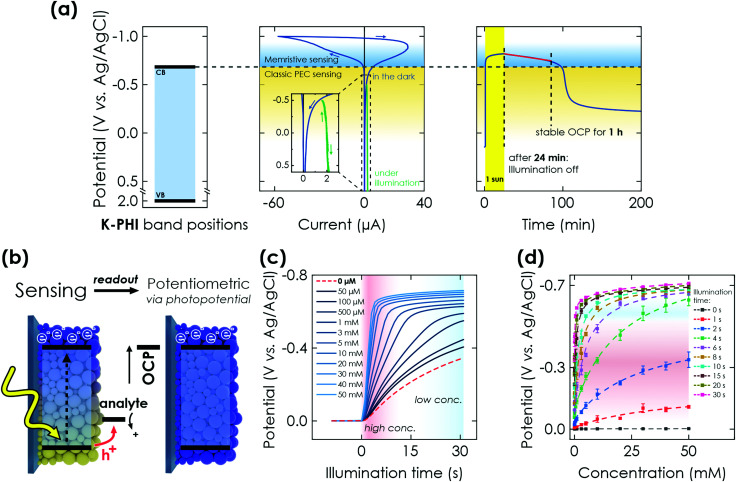
PEC properties of K-PHI and potentiometric memristive sensing *via* K-PHI films. (a) Left: Valence band (VB) and conduction band (CB) positions referenced to the Ag/AgCl reference electrode potential. Middle: Cyclic voltammetry (CV) measurement at 50 mV s^−1^, showing the behavior of K-PHI in the relevant potential window for sensing. At potentials more positive than −0.7 V *vs.* Ag/AgCl, a constant photocurrent is observed (green), which is used for direct PEC sensing. Charge storage occurs at potentials more negative than −0.7 V *vs.* Ag/AgCl. Right: Open circuit potential (OCP) evolution of K-PHI in the presence of glucose during illumination (photocharging) and its subsequent stability in the dark. (b) Scheme depicting memristive sensing and subsequent potentiometric readout *via* evaluation of the OCP. (c) Increase of the OCP during charging under illumination and in the presence of different glucose concentrations. With increasing concentrations, the OCP shift becomes more pronounced due to faster charging. (d) Correlation of the photopotential to the concentration of glucose. This concentration can be extracted at different illumination times independently, which allows to tune the sensitivity range. Dashed lines are non-linear fits of the sensor response over the entire dynamic. Fitting is discussed in Section S5.1 (ESI†).

Next, we demonstrate the photomemristive operation of the K-PHI sensor (Fig. 1(c)) based on the PEC sensing interaction outlined above. The necessary double functionality of charge storage and light absorption is illustrated in Fig. 2(a). Electric charging *via* cyclic voltammetry (CV) measurement shows the charging and subsequent discharging of the material occurring dominantly at potentials negative of −0.7 V *vs.* Ag/AgCl. Upon illumination with 1 sun (AM1.5 G) at open circuit potential (OCP) and in the presence of an oxidizable analyte, a photopotential immediately develops due to electron accumulation in this potential range, which remains stable for ∼1 h after 20 min of illumination (Fig. 2(a) right). The photopotential decay rate is very slow (1.21 mV min^−1^) and can be attributed to self-discharge *via* miniscule amounts of oxygen leaking into the reactor and being reduced by K-PHI (*e.g.*, ROS formation)^30,48^ or the FTO substrate promoting water reduction, since charge storage on K-PHI occurs at potentials more negative than the hydrogen reduction potential (*ca.* −0.61 V *vs.* Ag/AgCl at pH 7).^28^ Different analyte concentrations produce a different shift in this OCP (Fig. 2(c)). The inherent charge storage properties of K-PHI enable a quantitative comparison between the analyte concentration and the charging state of the material when the illumination time is fixed. The photomemristive behaviour integrates the analyte oxidation current and produces the stable change in the optoelectronic properties of K-PHI as the measurand. This accumulated charge is proportional to the charging current, which depends on both analyte concentration and illumination time. A longer illumination (or writing cycle) can improve the sensitivity for low analyte concentrations, as the system has more time to interact with the analyte and accumulate charges. In principle, there is no lower analyte detection limit in the absence of self-discharge (and other experimental limitations such as parasitic oxidation reactions due to a lack of selectivity), as the illumination time can be increased at will. On the other hand, a short light pulse allows for fast sensing of high analyte concentrations. Thus, K-PHI can act as receptor and transducer (as discussed in the previous paragraph) but also as a memristive amplifier due to its current integration mechanism, hence combining all sensor components on a single material. While related, it is important to contrast this sensing concept with persistent photoconductivity effects that have been reported in semiconductors like ZnO when exposed to gas molecules such as oxygen as the analyte.^49,50^ Whereas hole-induced desorption of chemisorbed oxygen leads to the accumulation of photogenerated electrons in the conduction band and their subsequent re-trapping by surface-adsorbed oxygen, electron trapping in K-PHI is mediated by the photointercalation of electrolyte ions into the bulk of the material, and is hence akin to memristors.^38^ The sole purpose of the analyte here is to act as a sacrificial electron donor (SED). In the following, three electrochemical readout methods of the sensor state are demonstrated and explained.

#### 2.2.1. Potentiometric readout

Potentiometric sensing at open circuit condition (OCP) evaluates the photopotential that a given K-PHI nanosheet thin film reaches after illumination with a fixed duration and intensity (1 sun), in the presence of a specified analyte concentration (Fig. 2(b)). After activation of the sample (Section S3, ESI†), upon illumination and in presence of the glucose in different concentrations, the OCP shifts negatively over time (Fig. 2(c)), which shows that the sensor is being charged. With increasing analyte concentration, the OCP response becomes more pronounced (shifts faster and to more negative potentials). In all cases, we observe a nearly linear regime until approximately −0.7 V *vs.* Ag/AgCl, followed by a second and significantly slower saturation regime. The gradient of the OCP shift depends on the differential charge density or capacity of K-PHI (see CV in Fig. 2(a)), which is comparably small before reaching −0.7 V *vs.* Ag/AgCl and primarily due to surface capacitance,^28^ followed by a faradaic response with a higher differential capacitance. The different charging kinetics can be exploited by using different illumination times to sense different effective analyte concentration ranges, thereby adapting the material's response window and hence, relative sensitivity. This becomes more evident when looking at the OCP evolution with increasing illumination times (Fig. 2(d)): at short illumination times of 1–4 seconds (red shading in Fig. 2(c and d)), higher glucose concentrations (10–50 mM) yield the most significant change in potential. Higher analyte concentrations can be measured more precisely as the OCP does not reach a value more negative than −0.7 V *vs.* Ag/AgCl, *i.e.*, no saturation occurs yet. When illuminating longer than 4 seconds (blue area in Fig. 2(c and d)), more charge accumulates over time at every concentration, which induces a stronger potential change. Hence, a steeper slope for small concentrations of 1–5 mM can be seen in Fig. 2(d), which enables more accurate sensing of low analyte amounts due to the extended interaction and memristive integration times. The illumination time can be used as a parameter to adapt the sensitivity of this potentiometric sensor (see Table 1). In principle, similar adjustments are possible by changing the light intensity and hence, photon flux, which generates the photoresponse. To extract the sensing information, a relationship between the sensing signal (photopotential) and analyte concentration is required. We have performed both phenomenological non-linear fitting and linear fitting of the sensor's response. The non-linear fitting (see Section S5.1, ESI†) uses an equation that mimics the contributions of the charging mechanism including their saturation, without requiring dedicated input parameters for a given set of conditions. With this non-linear approach, extracting the response of all electrochemical and optical readout modes over the entire concentration range is easily possible, with a much better fitting quality compared to the linear fit. A detailed description of the fitting is given in Section S5.1 (ESI†). The linear ranges are given in Table S1 (ESI†) and the fit is shown in Fig. S9 (ESI†). The non-linear fit is shown in Fig. 2(d).

**Table 1:** Summary of readout methods. A summary of recommended operation modes as well as key advantages that our memristive sensing concept provides in comparison to PEC amperometric sensing. Note that all memristive methods are tuneable *via* illumination time (as demonstrated for potentiometric sensing) or illumination intensity. Furthermore, combining measurement methods allows to combine their advantages, minimize the errors and improve accuracy.

Method	Useful range (glucose sensitivity in reported conditions)	Illumination time	Readout time	Sensing characteristics
Amperometric	0.1 mM to 10 mM (11 μM)	Continuous	60 s	– Direct sensing
Potentiometric	0.05 mM to 50 mM (50 μM)	<1 mM : >10 s	Instant	– Non-invasive
		1–10 mM : 4–10 s		– Memory sensing
		>10 mM : <4 s		
Impedimetric	0.05 mM to 20 mM (50 μM)	Only 30 s illumination analysed	<10 s to 100 s	– Non-invasive
				– Memory sensing
				– No fitting required
Coulometric	5 mM to 50 mM (100 μM)	Only 30 s illumination analysed	<300 s	– Memory sensing
				– Sensor reset (invasive)
Colorimetric	0.2 mM to 50 mM (200 μM)	Only 200 s illumination analysed	<1 s	– Minimum-invasive
				– Memory sensing
				– Wireless sensing
				– Visible colour change
Fluorometric	0.2 mM to 50 mM (200 μM)	Only 200 s illumination analysed	<1 s	– Minimum-invasive
				– Memory sensing
				– Wireless sensing

#### 2.2.2. Impedimetric readout

**Fig. 3:**
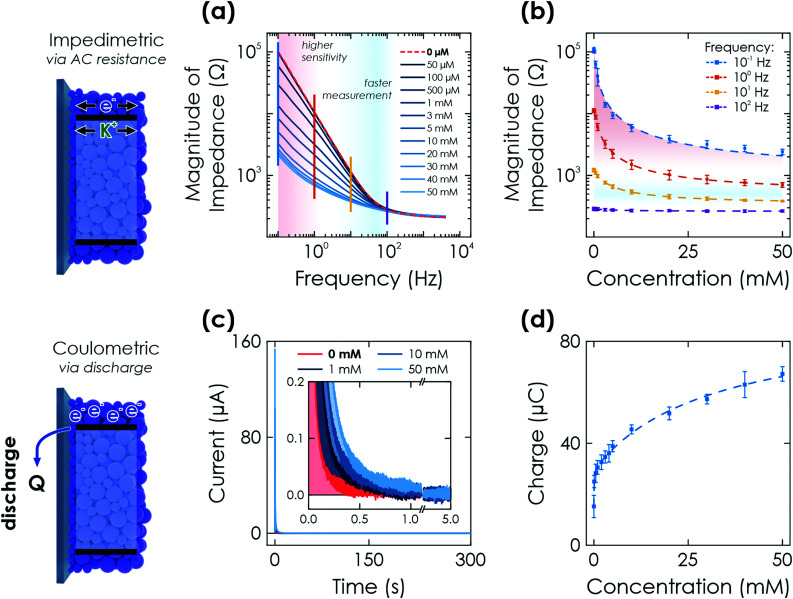
Further electrochemical readout methods *via* K-PHI thin films. (a) Impedimetric readout. Bode plot of the impedance response after illumination of the sensor for 60 s at OCP conditions and in presence of different glucose concentrations. (b) A change in magnitude of impedance can be correlated to the analyte concentration due to conductivity changes upon memristive charging. This effect has a varying signal amplitude at different time scales (*i.e.*, frequencies; shown with vertical lines in (a)) and concentrations. (c) Coulometric readout. Evolution of discharge current with time when applying a bias of +0.2 V *vs.* Ag/AgCl after illumination of the sensor for 60 s at OCP conditions and in presence of different glucose concentrations (inset shows a zoom of low currents). (d) The extracted charge Q correlates monotonously with the glucose concentration. Dashed lines in (b) and (d) are non-linear fits of the sensor response over the entire dynamic range. Fitting is discussed in Sections S5.2 and S5.3 (ESI†).

Impedimetric sensing utilizes electrochemical impedance spectroscopy (EIS) and probes the change in impedance (resistance) of the charged state, which is equivalent to a direct readout of the memristive state. The measurements are performed after illumination with 1 sun for 30 s at OCP by a 10 mV AC perturbation signal to extract the sensor's impedance. These measurements are non-invasive to the stored concentration information. When increasing the analyte concentration and hence, charge accumulation on K-PHI analogous to the potentiometric sensing discussed above (Fig. 2(c)), a decrease in magnitude of the impedance is measured and shown *via* a Bode plot in Fig. 3(a). We interpret this as the material becoming more conductive (less resistive) in response to charge accumulation of mobile charge carriers (photogenerated electrons and intercalated ions interacting with the backbone, equivalent to photodoping),^28,38,43^ as a consequence of the interaction with the analyte. The resistance can be interpreted as direct measure of the memristance (RM) of the system as it directly and monotonously relates to the charged state. The systematic relationship between the magnitude of the impedance and analyte concentration at different frequencies is shown in Fig. 3(b). In the frequency region <100 Hz, a change in impedance is observed that contains the sensing information. At frequencies <1 Hz (red shading in Fig. 3(a and b)), the most pronounced shift with respect to the analyte concentration is observed, which causes a steeper slope for both small and large concentrations. We attribute this low frequency range to the comparably slow faradaic charge storage process being triggered. It is kinetically slower than the double layer capacitance and therefore more visible at lower frequencies. The advantage of measuring at moderately higher frequencies (1–100 Hz) is the shorter measurement time (blue shading in Fig. 3(a and b)). However, for large analyte concentrations the difference in magnitude of impedance for different concentrations becomes less pronounced and is smaller than the measurement error, *i.e.*, it is not reliable. A realistic output can only be expected for <20 mM and frequencies of <10 Hz. Note that no impedance fitting is necessary as the concentration information can be extracted at a single frequency. Since the sensing interaction is analogous to potentiometric sensing discussed above, *i.e.*, charging under OCP conditions for a given time, sensitivity and dynamic concentration range can again be tuned by the illumination time and light intensity. Time delayed readout (‘memory’ sensing) is therefore possible as well.

We perform fitting of the relationship between impedance magnitude and analyte concentration with either a non-linear phenomenological fit over the entire dynamic range (Fig. 3(d) dashed lines and Fig. S10, ESI†) or linear with two linear ranges (0–1 mM and 10–50 mM, see Fig. S11, ESI†), albeit with significantly reduced fitting quality. A detailed discussion of the fitting is given in Section S5.2 and summarized in Table S1 (ESI†).

#### 2.2.3. Coulometric readout

Coulometric sensing quantifies the concentration of the analyte by discharging the sensor after charging it for a fixed time (here 60 s) after illuminating under OCP conditions in a degassed electrolyte containing a fixed glucose concentration. This readout should be distinguished from the others, as it is invasive, *i.e.*, it modifies the concentration information of the sensor during discharge. We perform the discharge by applying a constant potential of +0.2 V *vs.* Ag/AgCl and measuring the dark current. We observe an initial large current of up to ∼150 μA, which rapidly decays and plateaus after approximately 60 s (Fig. 3(c)), at which already 96% of the accumulated charges are discharged. We explain this fast decline with a decreasing amount of charge carriers available on the material and hence increasing resistance when discharging, a typical phenomenon for batteries,^51^ and in-line with the discussed correlation between resistance (*i.e.*, magnitude of impedance) and amount of charging above for impedimetric sensing. After 300 s of discharge, the average current for all experiments (irrespective of analyte concentration) was below 10 nA, which indicates a near complete discharging process. By integrating the current over the entire 300 s, we obtain a measure of the total charge that was stored on the system. This charge contains the sensing information (Fig. 3(d)), as it mirrors the amount of charging and thus, glucose concentration which had led to charge accumulation on K-PHI at a fixed illumination time as discussed above for potentiometric sensing (Fig. 2(c)). The sensitivity can again be tuned by appropriately fixing the illumination time and/or intensity during charging: short illuminations are beneficial for large concentration and long illuminations for low concentrations.

Note that this experiment also acts as a reset of the sensor for all memristive cases so that it can afterwards be reused for the next sensing experiment, *i.e.*, it deletes all previously stored information. This underlines that the change in memristive state is only dependent on the charged state and fully reversible, an important characteristic of a memristor.^52^

Combining this sensing method with a possible long charge retention time (when illuminating for 20 min, more than 1 h is possible; see Fig. 2(a)) allows us to demonstrate the delayed sensor readout, *i.e.*, ‘memory’ sensing. When charging the sensor for 60 s and performing the discharge after delays of 60 and 300 s, a decrease in charge is observed when increasing the delay time (see Section S6.1 for details, ESI†). We attribute this to self-discharge *via* uncovered parts of the FTO substrate due to water reduction from the aqueous electrolyte and oxygen reduction from oxygen leaking into the reactor. This self-discharge is also responsible for larger error bars in the signal to concentration correlation compared to the other electrochemical readout methods as due to the integrating mechanism, small differences among samples such as coating degree and density play a much more significant role. However, since sensor fabrication can be scaled easily (dip coating) and sample batches can be calibrated with an offset factor, these effects are of little practical concern (see Section S4, ESI†).

Fitting of the measurement to extract the concentration information is done with the same fit function as for the potentiometric readout over the entire concentration range (Fig. 3(d) dashed line) or by a linear fit that requires two linear ranges (0.1–5 mM and 10–50 mM, see Fig. S13, ESI†) with a much reduced fit quality. The delay times for memory sensing produce a systematic feature by either changing fit contributions or an offset for non-linear and linear fitting, respectively. An offset factor can thus be calculated to account for the self-discharge losses. Details of the fitting are given in Section S5.3 and Table S1 (ESI†).

### 2.3. Photomemristive sensing with K-PHI wireless particles

Finally, we study the optical response of K-PHI nanoparticles suspended in an aqueous suspension. This sensing mode is more direct and its implementation is potentially simpler compared to the previous ones, as no wires or electrode substrates are needed for *in situ* probing. The memristive charge accumulation is caused by the same light-induced oxidative charging process and reflects the accumulated sensing information *via* a change in photophysical properties.

#### 2.3.1. Colorimetric readout

**Fig. 4:**
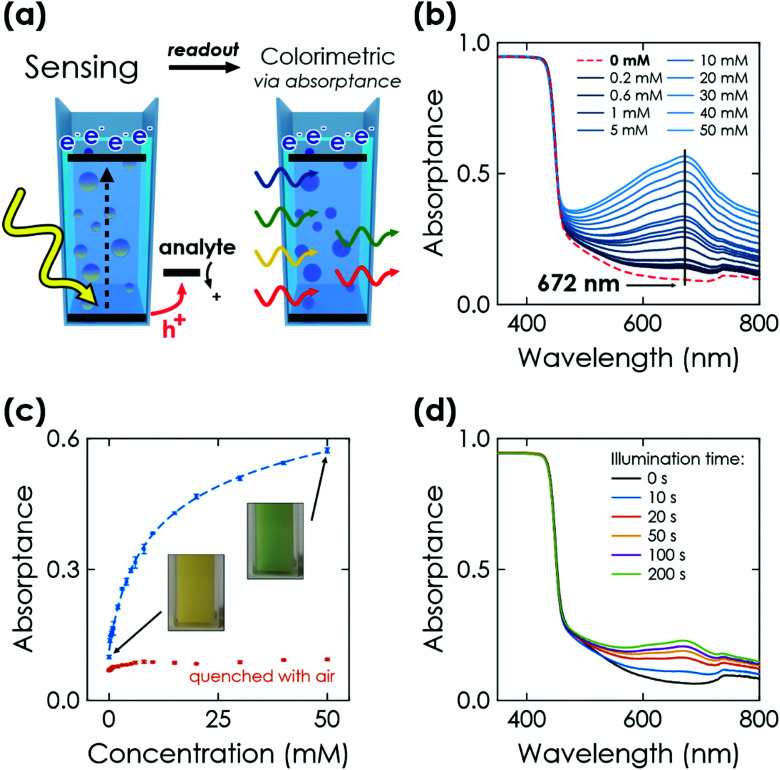
Colorimetric sensing *via* K-PHI wireless particles. (a) Scheme of memristive sensing and subsequent colorimetric readout. (b) Change of the absorptance at 672 nm after illuminating K-PHI particles suspended in an aqueous electrolyte for 200 s in presence of the example analyte glucose. With increasing concentrations, the absorption band with a maximum at 672 nm increases. (c) Correlation between the absorptance and glucose concentration. The red dots represent absorptance after quenching the respective charged state of K-PHI with air after the absorptance measurement. The colour change is visible by eye (inset pictures). (d) Dependence of illumination time on change of absorptance at a fixed glucose concentration of 1 mM.

The most prominent change in the photophysical properties of K-PHI due to photocharging is a colour change from yellow to blue, the so-called ‘blue state’.^24,26,42^ Measuring the K-PHI absorption after the sensing interaction allows to quantitatively relate the optical properties to the analyte concentration (Fig. 4(a)) in suspensions with a given concentration of K-PHI particles (3 mg mL^−1^). After photocharging for 200 s in the absence of oxygen and in the presence of the analyte (here glucose), the absorptance of the suspension was measured from 800 to 350 nm (Fig. 4(b)). Note that for higher accuracy, absorptance and not absorbance is measured in an integrating sphere, to correct for scattering. With increasing analyte interaction, an additional absorption band with a maximum at ∼670 nm appears and grows, which is characteristic for the ‘blue state’.^24^ Control experiments without glucose confirmed no absorptance at wavelengths above the optical bandgap of K-PHI (∼450 nm). Plotting the value of the absorptance at this maximum against the analyte concentration reveals the colorimetric sensing ability (Fig. 4(c)). An increase in absorptance is detectable down to a glucose concentration of 200 μM for the chosen illumination time and K-PHI concentration. We have also investigated influences, which stem from the particle size. Although a slight offset could be observed (see Section S7.1, ESI†), no systematic change was found.

Similar to the above-discussed electrochemical readout methods, illumination time can be used to tune the response towards a more specific sensitivity range (Fig. 4(d) and Section S7.2, ESI†). Furthermore, a time-delayed readout of the sensing information is easily possible, with a signal decay of ∼30% after a delay of 20 min (see Section S7.3, ESI†). This enables for example *in situ* measurements with *ex situ* readout in environments where *in situ* optical sensing is not possible. Note that the colour change is also clearly visible by eye (Fig. 4(c), inset), which makes this readout method useful for label-free colorimetric applications and qualitative analysis even without instrumentation. In comparison to previously reported colorimetric carbon nitride sensors that rely on colour changes of external species such as 3,3′,5,5′-tetramethylbenzidine (TMB), our approach does not require any additional external signal molecules to achieve a visible colour change, minimizing fabrication cost and time while simultaneously improving the design simplicity and recyclability.^33^ The sensing information can be erased by opening the cuvette and enabling quenching of the reduced state by oxygen contained in air.^28,30^ Notably, the quenched state always restores the initial absorptance value (Fig. 4(c), red dots) and no degradation of the material's optical properties was observed. This resetting method is more practical than washing the sensor to reset the concentration of external species (such as TMB). The relationships between absorptance signal and concentration can be fit analogous to the above discussed electrochemical readout methods. We find three linear ranges (0.2 to 2 mM, 3 to 10 mM, 15 to 50 mM, see Fig. S15, ESI†) with a limited fitting quality. Phenomenological non-linear fitting works much better and over the entire dynamic concentration range (Fig. 4(c) dashed line and Fig. S14, ESI†). A more detailed discussion of the fitting is given in Section S5.4 and summarized in Table S1 (ESI†).

#### 2.3.2. Fluorometric readout

**Fig. 5:**
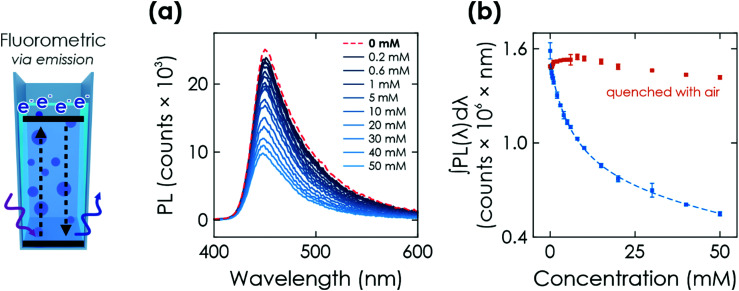
Further optical sensing method *via* K-PHI wireless particles. (a) Fluorometric readout. Photoluminescence (PL) emission when exciting the suspended K-PHI particles at a wavelength of 370 nm after illumination for 200 s in presence of different glucose concentrations. The PL quenching is caused by accumulated charges. (b) The red dots represent PL emission after quenching the respective charged state of K-PHI with air after the PL measurement.

When exciting the characteristic and broad fluorescence emission of K-PHI at 370 nm, the emission signal with a maximum at ∼450 nm can also be used to characterize the charged state, analogous to the colorimetric measurements. After illuminating the K-PHI nanoparticle suspension with different glucose concentrations for 200 s, we measured the emission spectrum from 400 to 600 nm (Fig. 5(a)). With linearly increasing glucose concentrations, we observed a fast-decreasing fluorescence activity (Fig. 5(b)). Since the absorption at the excitation wavelength (370 nm) remains unchanged (Fig. 4(b)), this decline is purely an emission property, which is caused by the accumulation of electrons, enabled by hole extraction due to glucose oxidation. We attribute the emission quenching to an increased recombination probability of photogenerated holes, which have more recombination partners with increasing amounts of electrons being stored on the material. This de-excitation, induced by accumulated charges, may be either radiative at frequencies out of our detection limits (>900 nm) or non-radiative. A schematic summary of this model together with a discussion of the negligible invasiveness of these measurements is given in Sections S7.4 and S7.5 (ESI†). The glucose concentration cannot only be quantified by integrating the emission spectrum (Fig. 5(b)), but also by an emission intensity measurement at a single wavelength. By that, the measurement time can be significantly decreased (20 s to 0.1 s). The sensitivity of the fluorometric readouts is comparable to the colorimetric readout, *i.e.*, a change in signal can be detected down to 200 μM for the chosen illumination time of 200 s. Emission from the quenched sensor (Fig. 5(b), red dots) without washing the material or exchanging the electrolyte reveals a slight decay in the emission of the discharged state, which we attribute to slow particle agglomeration or surface clogging by oxidized donor species.^46^ Both can be restored by washing away the analyte and its oxidation products, or by sonicating the material in order to fully restore the properties of the sensor material.^46^ Besides, a slight dilution of K-PHI suspension due to the addition of glucose solution might also contribute to the signal decay. The relation between the analyte concentration and the signal can be fit analogous to the colorimetric readout, with three linear ranges (0.2 to 2 mM, 2 to 10 mM, 15 to 50 mM; see Fig. S17, ESI†) or one non-linear fit over the entire dynamic concentration range (Fig. 5(b) dashed line and Fig. S16, ESI†). A detailed discussion of the fitting is given in Section S5.5 and Table S1 (ESI†). Last and similar to before, the sensitivity ranges can be tuned by varying the illumination time (Fig. S20, ESI†) or intensity and also by increasing the power of the laser for photoluminescence excitation. Besides, also time-delayed readout is possible with a signal loss of only ∼20% after a delay of 1200 s (see Section S7.3, ESI†), caused by oxygen leaking or – in principle – other electron acceptors.^30,48^ In principle, the wireless methods described here are applicable also to K-PHI particles immobilized on a thin film. However, the application of wireless K-PHI particles for sensing holds more promise for applications where environmental or biological conditions are studied *in situ*.^48^

## 3. Conclusion

We have introduced the biocompatible, environmentally friendly K-PHI as a photomemristive sensing platform enabling multimodal analyte sensing. Building on the classical PEC amperometric sensing approach, in which an oxidative photocurrent can be correlated to the concentration of various analytes, we develop a photomemristive sensing concept, which utilizes the material's inherent bifunctionality of light absorption and charge storage to photo-oxidatively sense and quantify analytes by accumulating the generated electrons on the material. The change in the material's optoelectronic properties upon photocharging translates into various electrochemical and optical readout methods: (i) potentiometric and (ii) impedimetric sensing (non-invasive), (iii) coulometric sensing (invasive and method to reset), as well as (iv) colorimetric and (v) fluorometric sensing (minimally invasive). The latter two optical methods enable wireless analyte quantification for *in situ* applications as particulate systems and a facile qualitative visual readout.^30,48,53^ Contrary to most other sensors, the sensing methods are all direct, *i.e.*, we combine receptor, transducer and current integrating memristive amplifier on the same material, while no additional intermediate species (*e.g.*, mediators) are required. In comparison to state-of-the-art memristive sensors requiring tailored nanostructures, the K-PHI sensor is simple (only a degassed standard electrochemical cell is required), flexible (nanosheet film electrodes or wireless sensing of nanoparticles) and easily scalable (cheap and easy material synthesis, film preparation *via* solution process). Moreover, the sensor can be reset and easily reused. Input parameters for sensing such as illumination time and intensity can be used to tune the sensitivity and measuring ranges. A time-delayed readout is possible. The main practical advantage of our sensing approach is its great flexibility for different applications, which enable fast (<1 s) and facile readout, as well as the combination of different techniques for adapting sensitivity ranges without modification of the sensor. A summary of the key advantages and measurement properties of the different analysis methods is given in Table 1. The organic nature of K-PHI with functionalizable groups at its edges was further shown to enable a targeted surface property engineering by chemical modifications, which can lead to much enhanced selectivity.^46^ Similarly, the chemical attachment of dedicated receptors appears possible,^54^ which would allow to engineer selectivity and responsivity further, according to the field of application. Besides sensing, the concepts described here are useful for evaluating SED strengths and thereby, to provide a better understanding of photocatalytic process parameters, while also being of interest as a characterization tool for light-driven charge storage properties of ‘solar battery’ materials. At the same time, these novel sensing approaches can be applied to other photocatalytic and especially light-storing materials, including both organic and inorganic semiconductors. Since the memristive sensor generates an electrical signal upon charging (OCP), which can be used to drive a current by discharging (coulometric measurements), it can be coupled to other (bio)electronic devices in feedback loops, which can be triggered by using voltage or current thresholds. With this, true neuromorphic applications that facilitate automated electronic signal processing would be directly enabled.^4,5,12,55,56^

## 4. Experimental

### 4.1. Synthesis of the carbon nitride modification K-PHI as nanoparticles

The carbon nitride materials were synthesized as described in literature.^24,26,36^ The precursor material melamine as well as potassium thiocyanate were acquired from Sigma Aldrich in reagent grade purity. Exfoliation was carried out in 2-propanol (IPA) *via* sonication in an ice bath for 2 h (300 mg K-PHI in 100 mL IPA). Subsequently the nanosheets were separated *via* two centrifugation steps at 353 RCF for 20 min and 795 RCF for 40 min in a centrifuge (3–30k, Sigma) to ensure a uniform small particle size, akin to a reported procedure.^27^ To reach the desired concentration, density was first evaluated by drying 1 mL of suspension and measuring the weight of the dried residue on a quartz crystal microbalance. To increase the particle concentration to 0.2 mg mL^−1^ excess IPA was then removed using a rotary evaporator (Hei-Vap Value Digital G3B, Heidolph) at a pressure of 137 mbar and a water bath temperature of 50 °C.

### 4.2. Material characterization

ATR-IR spectra of K-PHI bulk suspensions and nanosheets were collected with an IR spectrometer (UATR TWO, PerkinElmer), which was equipped with a diamond crystal. The optical bandgap of K-PHI bulk suspensions and nanosheets was characterized with an UV-VIS spectrometer (Cary 5000, Agilent), equipped with an integrating sphere. Films were characterized *via* AFM (MFP-3D, Asylum Research), SEM (ZEISS Merlin electron microscope), and TEM (CM 30 ST (300 kV, LaB6 cathode, Philips)).

### 4.3. Preparation of the sensor films and suspensions

Thin films of K-PHI nanoparticles were deposited onto FTO substrates (Sigma Aldrich, surface resistivity of 7 Ω cm^−2^) *via* dip coating with 400 dips, 100 mm min^−1^ extraction speed and 120 s drying time at ambient temperature between the dips (ND-R Rotary Dip Coater, Nadetech). A subsequent annealing at 70 °C for 2 days was performed to ensure removal of all leftover solvent. The sample was then cut into 10 × 12 mm and a small part of the film was scratched off for contacting. This was performed by gluing a wire to an uncovered part of the FTO using conductive silver paste (Silver Conductive RS 186-3600, RS-Pro). The contact was then sealed with epoxy glue (DP410, 3 M Scotch-Weld) to provide a rigid connection and prevent both the silver paste and uncovered FTO to influence the measurements. Last, an electrochemical self-cleaning of the finished samples was performed according to a procedure described in Section S3 (ESI†). For measurements, which utilize particles in a suspension, dried as synthesized bulk K-PHI was suspended in water (3 mg mL^−1^) and vortexed for 120 s to ensure a proper distribution of particles.

### 4.4. Electrochemical measurements

All electrochemical measurements were performed in an aqueous electrolyte which contained a 10 mM KCl (Sigma Aldrich) background electrolyte. The electrolyte was purged with >99% argon for at least 20 min through a porous glass frit before every measurement. An oxygen content of <100 ppb during measurements was ensured by measuring trace oxygen with a trace optical oxygen meter (PSt6 sensor spot and Fibox 3 trace, Presens). All analytes (Sigma Aldrich) were dissolved in deionized (DI) water and added to the electrolyte in respective concentrations. An Ag/AgCl electrode with saturated KCl electrolyte (RE-1CP, ALS Japan) was used as the reference electrode and a gold foil (Sigma Aldrich) as counter electrode. Measurements were carried out with a multichannel potentiostat (Autolab M204, Metrohm) in a glass reactor equipped with a quartz window for illumination. Impedance measurements were carried out with a single-channel potentiostat (CompactStat, Ivium). Impedance fitting was performed with the RelaxIS 3 software, rhd Instruments.

Illumination (1 sun) was generated by a calibrated Sciencetech LightLine A4 solar simulator, which provides simulated sunlight with class AAA quality (AM1.5G). Light was turned on and off using a ThorLabs SHB1T shutter.

### 4.5. Optical measurements

All optical measurements were performed in a quartz cuvette (Hellma Analytics). The suspension (K-PHI in DI water, 3 mg mL^−1^) was purged with >99% argon before every measurement for 300 s and during charging illumination. Glucose (Sigma Aldrich) was dissolved in DI water and added into the cuvette in respective concentrations. The sample was illuminated (AM1.5 G) using a solar simulator (IEC/JIS/ASTM, Newport). Fluorescence was measured using a spectrofluorometer (FLS980, Edinburgh Instruments). Absorptance spectra were measured using a spectrophotometer equipped with an integrating sphere (Cary 5000 UV-VIS, Agilent). The sample was positioned in the centre of the sphere on an angle to obtain both total transmission and total reflection signals.

## Author contributions

AG, AJS, FP and BVL conceived the project. AG performed the electrochemical measurements. AG and AJS performed the optical measurements. AG and AJS, with assistance of FP, analysed the data. AJS, NVB, FP and BL supervised the research. AG, AJS and FP wrote the manuscript with assistance of all authors.

## Conflicts of interest

The authors declare no conflict of interest.

## Supplementary Material

MH-009-D2MH00069E-s001
