# Supplementary material for: Photomemristive sensing via charge storage in 2D carbon nitrides
Source: Mater Horiz. 2022 Apr 26;9(7):1866–77. doi: 10.1039/d2mh00069e (PMC9252257; doi:10.1039/d2mh00069e)
Supplement: MH-009-D2MH00069E-s001 [file MH-009-D2MH00069E-s001.pdf]

Electronic Supplementary Material (ESI) for Materials Horizons.
This journal is © The Royal Society of Chemistry 2022

Supporting Information for:

Photo-Memristive Sensing via Charge Storage in 2D Carbon Nitrides

Andreas Gouder,[a,b] Alberto Jiménez-Solano,[a] Nella M. Vargas-Barbosa,[c] Filip Podjaski,[*a] and Bettina V. Lotsch*[a,b]

[a.] Department Nanochemistry, Max Planck Institute for Solid State Research, Heisenbergstr. 1, 70569 Stuttgart, Germany.
E-mail: f.podjaski@fkf.mpg.de, b.lotsch@fkf.mpg.de
[b.] Department Chemistry, Ludwig-Maximilians-University, Butenandtstr. 5-13, 81377 Munich, Germany.
[c.] Institute for Energy and Climate Research (IEK-12), Helmholtz Institute Münster, Forschungszentrum Jülich, Corrensstr. 46, 48148 Münster, Germany.
* Corresponding authors.

Contents

1. Material and thin film analysis 2
 1.1. Molecular structure and optical analysis 2
 1.2. Structure Analysis of K-PHI Films 2
2. K-PHI as a direct amperometric PEC sensor 4
3. Electrochemical self-cleaning of thin films 5
4. Normalizing coulometric readout measurements 6
5. Fitting of the sensor curves 6
 5.1. Potentiometric sensor 9
 5.2. Impedimetric sensor 13
 5.3. Coulometric sensor 15
 5.4. Colorimetric sensor 17
 5.5. Fluorometric sensor 19
6. Additional electrochemical analysis of memristive properties of K-PHI films 21
 6.1. Delayed coulometric sensor information readout 21
7. Additional optical analysis of memristive properties of K-PHI particles 22
 7.1. Influence of particle size 22
 7.2. Dependence on illumination time 23
 7.3. Delayed sensor information readout 24
 7.4. Negligible invasive nature of the optical memristive state readout 25
 7.5. Schematic representation of the PL characterization process by the quenched PL emission 26
8. References 27

1. Material and thin film analysis

1.1. Molecular structure and optical analysis

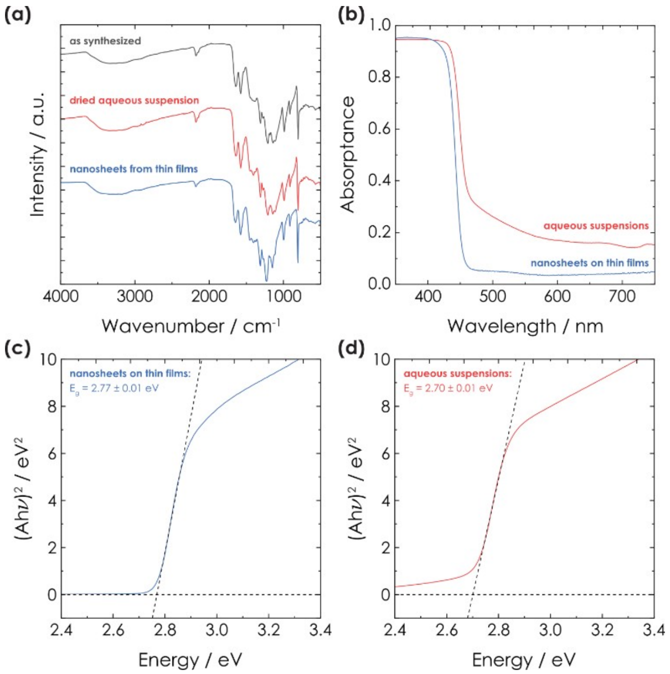

Figure S1. Structural and optical characterization. **(a)** FT-IR characterization of K-PHI as-prepared, as dried nanosheets from aqueous suspension and scratched off from K-PHI nanosheet films on FTO. The spectra are normalized to the characteristic heptazine vibration at 815 cm-1. No significant changes in the structure of the material are observed. The characteristic NCN-stretching-signal indicating possible hydrolysis at 2180 cm-1 for K-PHI does not change significantly.[1] **(b)** Absorptance spectra of K-PHI as nanosheets deposited onto FTO and aqueous suspensions (3 mg/mL). **(c)** and **(d)** Tauc plot of K-PHI nanosheets deposited onto a FTO film (c) and in an aqueous suspension (d). It is in agreement with the reported literature bandgap value of K-PHI.[2]

1.2. Structure Analysis of K-PHI Films

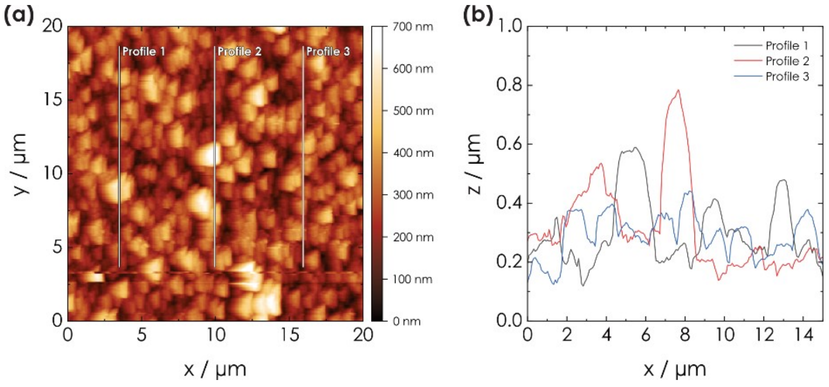

Figure S2. AFM Characterization of thin films. **(a)** Atomic force microscopy (AFM) image for a K-PHI thin film sensor. The surface has a rather large roughness with a root mean square (RMS) roughness of 110 nm. Three profiles were extracted for further analysis along the lines shown within the picture. **(b)** Height profiles alongside the lines shown in (a).

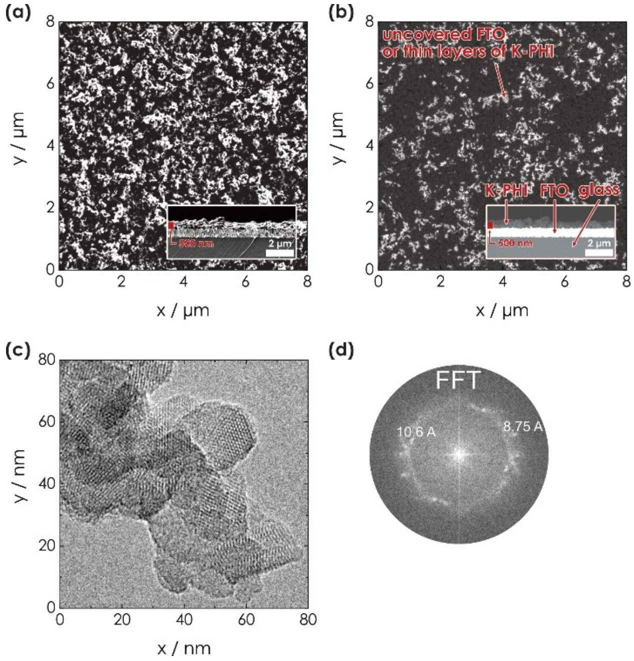

Figure S3. SEM and TEM Characterization. **(a)** SEM images for a K-PHI thin film sensor via the secondary electron detector showing irregular surface morphology below the µm-scale. The inset shows the cross section of the film via the same detector. **(b)** SEM image of the same spot shown in (a) visualized by the backscattered electron detector (ESB). The bright parts correspond to either uncovered FTO parts or thin layers of K-PHI through which the FTO is still visible, and the dark parts correspond to thicker layer areas. The inset shows again the cross section of the film (ESB detector). The FTO layer on top of the glass is clearly visible. The dark layer deposited on top of the FTO represents K-PHI. **(c)** TEM images of K-PHI particles, showing the contours of crystalline nanoparticles with lateral dimensions ranging from 20-60 nm. **(d)** FFT image of K-PHI particles shown in (c).

2. K-PHI as a direct amperometric PEC sensor

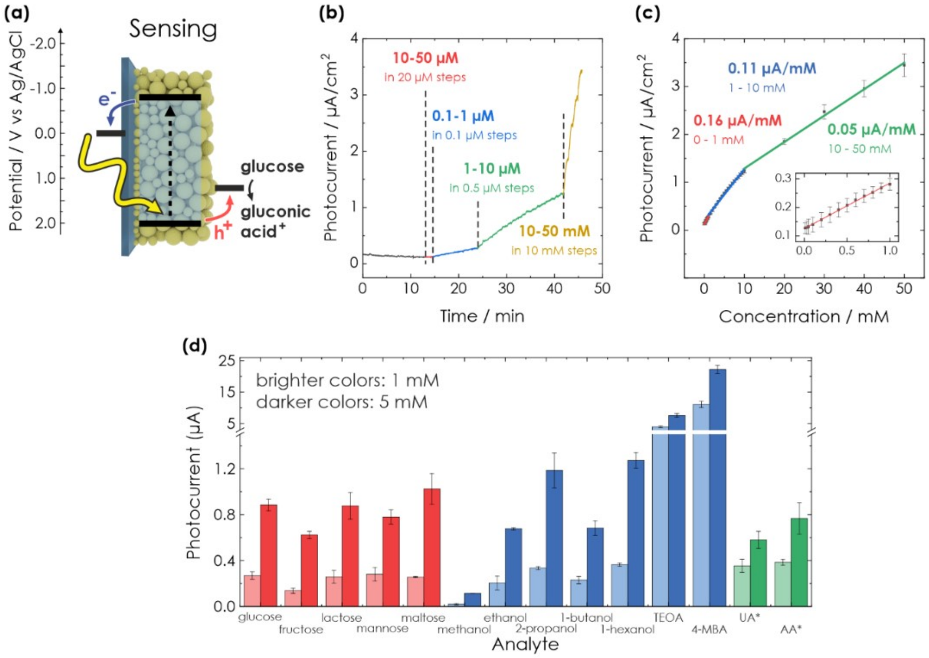

Figure S4. K-PHI as a direct amperometric PEC sensor. **(a)** Scheme of a classic oxidative PEC sensor, in which photoexcited charges generate current. Holes oxidize the analyte, the electrons are extracted continuously, mirroring the reaction rate. **(b)** Photocurrent of a K-PHI sensor under continuous illumination at a bias of 0 V vs. Ag/AgCl in a degassed 10 mM KCl solution with stepwise increasing glucose concentrations. **(c)** Relationship between photocurrent and concentration of glucose. Three linear ranges with a R2 > 0.99 are fitted to the two glucose concentration ranges 0 to 1 mM (inset, red), 1 to 10 mM (blue) and 10 to 50 mM (green). The limit of detection (LOD), 11.4 µM, was calculated via the smallest red concentration range (inset). **(d)** Photocurrent of a direct amperometric K-PHI sensor for different sugars (red), alcohols (blue) and the physiologically relevant molecules ascorbic acid (AA) and uric acid (UA) (green). Measurements are performed at a concentration of 1 and 5 mM for sugars and alcohols, and 0.1 and 0.5 mM for UA and AA with a bias of 0 V vs. Ag/AgCl, highlighting the versatility of K-PHI sensors.

We have investigated the ability of K-PHI thin films to perform direct photoelectrochemical amperometric sensing via the case study of glucose. The sample was immersed into a degassed aqueous electrolyte and activated (see SI Section 3 for details). Degassing is necessary to prevent charge losses due to side reactions such as the formation of reactive oxygen species (ROS).[3] A potential of 0 V vs Ag/AgCl is applied to provide a driving force for electron extraction. While glucose is added in stepwise increasing concentrations from 10 µM to 50 mM in 1 min cycles, the average photocurrent over the last 10 s (i.e. after initial stabilization) of each cycle is measured (Figure S4 (b)).[4] We observe a linear scaling of the photocurrent with glucose concentration for three distinct concentration ranges: 0-1, 1-10 and 10-50 mM, with average slopes of 0.16, 0.11 and 0.05 µA/mM, respectively (Figure S4 (c)). In the regime between 0 and 1 mM, a limit of detection (LOD) of 11.4 µM (0.21 mg/dL) can be determined.[5] Note, that in the main text we do not discuss the last largest slope, since the measurement errors are too large to gain a useful sensing information.

To underline the versatility of K-PHI amperometric sensors as a sensing platform, we have investigated other analytes, such as sugars (Figure S4 (c), red bars), alcohols (Figure S4 (c), blue bars) and other physiologically relevant molecules (Figure S4 (c), green bars). At a concentration of 5 mM, the mono- and disaccharide sugars (Figure 2 (a), red) produce photocurrents between ~0.6 and 1 µA/cm^2. By far the strongest response is observed for triethanolamine (TEOA) and 4-methylbenzyl alcohol (4-MBA) - both typical sacrificial electron donors for photocatalytic studies[2,6–8] - with photocurrents of 7.5 and 23 µA/cm^2, respectively. The physiologically relevant molecules ascorbic acid (AA) and uric acid (UA) were measured at lower concentrations of 0.1 and 0.5 mM, as usually found in blood serum.[9] A significant photocurrent response of 0.38 and 0.35 µA/cm^2 is observed already at a concentration of 0.1 mM for AA and UA, respectively.

3. Electrochemical self-cleaning of thin films

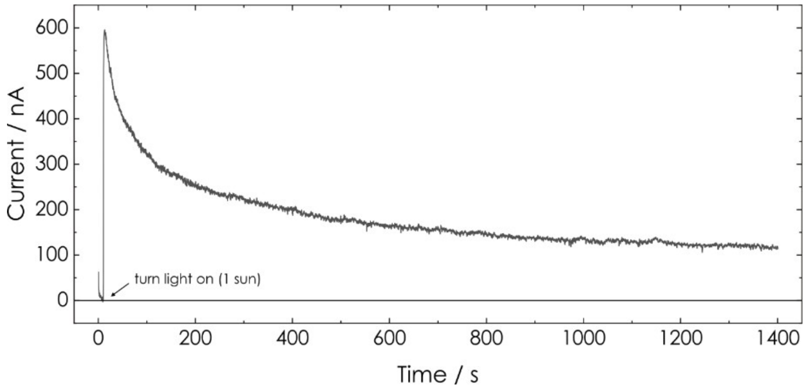

Figure S5. Pretreatment of K-PHI thin films by self-cleaning. Self-cleaning of a fresh K-PHI thin film in 10mM KCl. A bias potential of 0 V vs Ag/AgCl is applied and after 10 s the sample is illuminated with 1 Sun (AM 1.5G) until a current of 120 nA is reached.

The K-PHI thin films are very sensitive to organic contaminations as well as for charging through ambient light – per se beneficial properties for a sensor. However, it is essential to remove any unwanted potential sources of charges before starting the sensing experiments. Otherwise, those charges could influence the sensor output as they would contribute to the memristive change in material properties. Thin films of K-PHI for potentiometric and impedimetric sensing were thus first immersed into a degassed reactor without any analyte and a bias potential of 0 V vs Ag/AgCl was applied. Subsequently, the sample was illuminated until a current of < 120 nA was reached. The required illumination time to achieve this varied between 30 min and 120 min. An exemplary measurement is shown in Figure S5. The initial peak in the dark is caused by electrons stored onto the K-PHI film prior to applying a bias. The photocurrent that develops initially during the very first illumination could be linked to organic contaminations either from the reactor or the K-PHI thin film (e.g., residues of isopropanol from the thin film deposition). When those miniscule organic contaminations are oxidized and thus consumed, the photocurrent slowly decreases.

4. Normalizing coulometric readout measurements

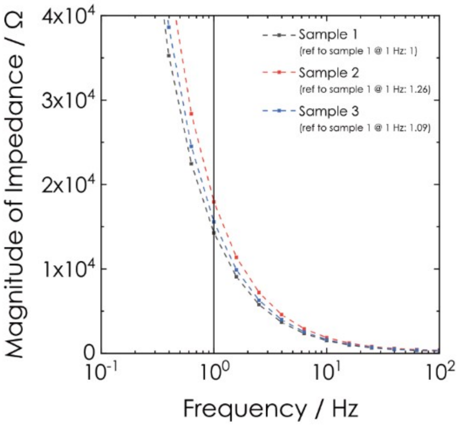

Figure S6. Reference impedance measurement for coulometric readout. Bode plot of the magnitude of impedance from the impedance measurement of the samples used for coulometric sensing after the sensing measurements. To calculate a correction factor for better comparison of the different samples, the magnitude of each sample at 1 Hz (vertical line) was normalized to the first sample. The correction factors are given in the legend.

As stated in the main text in Coulometric sensing, this method is very sensitive on K-PHI thin film properties such as size and film thickness as the readout is the integration of the current over the entire 5 mins of discharging. Thus, to allow a better comparison between samples, an impedance measurement was carried out after each measurement. This measurement is shown in Figure S6 for three samples for coulometric sensing. A small variation in magnitude of the impedance can be observed. To reference the measurements to each other, a correction factor was calculated and multiplied to the respective coulometric measurements. To do that, the magnitude of impedance of the impedance measurements at 1 Hz was normalized to the first sample. Thus, the correction factors for coulometric sensor samples discussed in the main text (denoted herein as sample 1, 2 and 3) are 1, 1.26 and 1.09, respectively (see legend in Figure S6).

5. Fitting of the sensor curves

For an application of our sensor, a fitting function has to be found that gives a numeric relation between concentration of glucose and the sensor output. In this section, we will discuss both linear and non-linear fitting of our different sensor methods. In Table S1, the results of all the fits in their respective ranges are summarized.

Linear fitting

Linear fitting allows a very easy and straightforward relationship between analyte concentration and sensor signal and is therefore typically used to fit sensors.[5,10] We have chosen linear ranges so that they reach a fit quality of $R^2 > 0.9$ (with two exceptions of $R^2 > 0.8$ as discussed below). The slope of the linear

range is a measure for how accurate the sensor can measure: With a steeper slope and hence, larger response factor, the measurand error (e.g., OCP for potentiometric sensing) has a smaller impact on the analyte concentration error, which is more desired for the application of our sensor.

Non-linear fitting

The sensor signal is not linear over its whole dynamic range, which is partially due to the non-linear charge storage characteristics.[11] Thus, either linearization of the sensor output via e.g., an external circuit or non-linear fitting can improve the accuracy of the sensor's signal.[5,10,12] Non-linear monotonous signal fitting can be adapted better to the sensor mechanism, thereby extending the dynamic sensing range. A biosensor system where the sensing response is governed by adsorption, can be fitted with the function:[13]

$$signal(x) = A_1 + \frac{B_1 \cdot x}{B_2 + x} \qquad \text{(S1)}$$

where $signal(x)$ is the measured sensor signal, x the analyte concentration and A_1, B_1 and B_2 are the fitting parameters. While A_1 gives the offset of the sensor signal when no analyte is present, B_1 takes into account the signal when all sensing sites on the sensor are occupied with the analyte.[13] B_2 relates to the dissociation constant of the analyte from the sensor.[13] Phenomenologically, this equation can approximate any linear behavior (B2 >> x) and well approximate exponential type behaviors (e.g., isotherms).

In case of our memristive sensor, we have both non-linear diffusion controlled (analyte diffusion to the sensor, faradaic charging process), linear (capacitive charging of the sensor), adsorption (sensor clogging) as well as other processes (e.g., conductivity limitations (especially when not charged), self-discharge). Furthermore, faradaic charging only occurs at potentials < -0.7 V vs. Ag/AgCl (see main text Figure 2(b)). Thus, it is difficult to assign specialized equations to specific processes. We therefore modified the very general equation (S1) to account for all above described processes to perform a phenomenological, which allows to extract the analyte concentration information from the sensing signal over the entire dynamic range.

We separate the description of the charging behavior into two major contributions: 1: A process which is governed by the diffusion of analyte to the sensor and capacitive charging. The latter only occurs at potentials > -0.7 V vs. Ag/AgCl. 2: A process consists mainly of the faradaic charging and therefore, only possesses a major contribution at potentials < -0.7 V vs. Ag/AgCl. Sensor clogging as well as conductivity limitations influence both processes. Hence, equation (S1) is modified accordingly:

$$signal(x,t) = A_1(t) + \frac{B_1(t) \cdot x}{B_2(t) + x} + \frac{C_1(t) \cdot x}{C_2(t) + x} \qquad \text{(S2)}$$

The process described with the coefficient $B_1(t)$ and $B_2(t)$ describe the capacitive charging and the process with $C_1(t)$ and $C_2(t)$ the faradaic charging. $A_1(t)$ gives the offset of the blind current when no analyte is present. All processes depend on the illumination time t. Furthermore, to correct for the non-linear distribution of measurement points at different analyte concentrations, the derivative of

concentrations (difference to the two neighboring analyte concentration values) was chosen as a weight for all fitting points.

Table S1. Summary of sensor fitting results. Two different fittings where performed for all sensor readout methods: Linear fitting and non-linear fitting according to equation (S2) from Section 4 above. Results of ranges as well as the fitting error R2 is given. The "*" marks values which are tunable via illumination time (as with potentiometric) or illumination intensity (herein everything measured with 1 sun).

<table>
<tr><th>Method</th><th>Illumination time</th><th>Linear ranges</th><th>Non-linear ranges</th></tr>
<tr><td>Amperometric</td><td>constant</td><td>0.1 mM – 10 mM</td><td>-</td></tr>
<tr><td rowspan="2">Potentiometric</td><td>1-2 s[a)]</td><td>0.05 mM[a)] – 50 mM[a)]
($R^2 > 0.93$)</td><td rowspan="2">50 µM[a)] up to 50 mM[a)]
(for > 1 s: $R^2 > 0.98$)</td></tr>
<tr><td>> 2 s[a)]</td><td>4 s: 0.05 mM[a)] – 20 mM
30 mM – 50 mM
6-8 s: 0.05 mM – 10 mM
20 mM – 50 mM
10 s: 0.05 mM – 5 mM
10 mM – 50 mM
>15 s: 0.05 mM – 1 mM
10 mM – 50 mM[a)]
(all $R^2 > 0.83$)</td></tr>
<tr><td>Impedimetric</td><td rowspan="2">30 s[a)]</td><td>0.05 mM[a)] – 1 mM
($R^2 > 0.97$)

10 mM – 50 mM[a)]
($R^2 > 0.82$)</td><td>50 µM[a)] to 50 mM[a)]
($R^2 = 0.99$)</td></tr>
<tr><td>Coulometric</td><td>0.1 mM[a)] – 5 mM
($R^2 > 0.93$)

10 mM – 50 mM[a)]
($R^2 > 0.97$)</td><td>100 µM[a)] to 50 mM[a)]
($R^2 > 0.99$)</td></tr>
<tr><td>Colorimetric</td><td rowspan="2">200 s[a)]</td><td>0.2 mM[a)] – 2 mM
($R^2 = 0.97$)

2 mM – 10 mM
($R^2 = 0.98$)

20 mM – 50 mM[a)]
($R^2 = 0.99$)</td><td>200 µM[a)] – 50 mM[a)]
($R^2 = 0.99$)</td></tr>
<tr><td>Fluorometric</td><td>0.2 mM[a)] – 2 mM
($R^2 = 0.99$)

2 mM – 10 mM
($R^2 = 0.99$)

15 mM – 50 mM[a)]
($R^2 = 0.98$)</td><td>200 µM[a)] – 50 mM[a)]
($R^2 = 0.99$)</td></tr>
</table>

[a)] further tunable with illumination intensity and/or time

5.1. Potentiometric sensor

Non-linear fitting

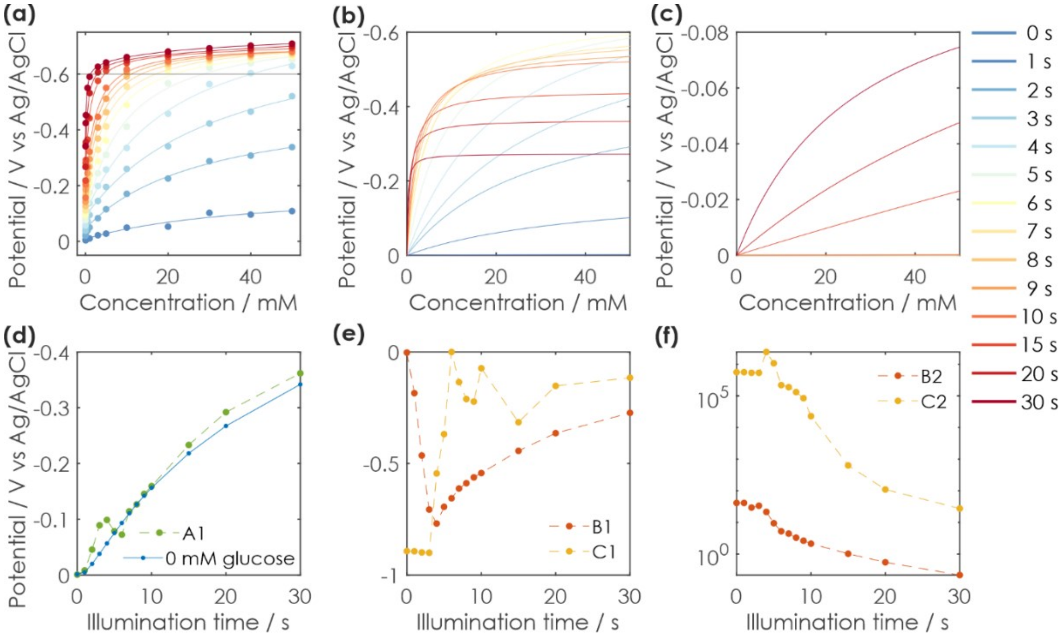

Figure S7. Non-linear fitting of the potentiometric sensing measurement. **(a)** The OCP change with glucose concentration is plotted after different illumination times. This measurement is discussed in the main text Potentiometric sensing. Fitting of the results was performed according to equation (S2) in the main text. This Figure is a reproduction of Figure 3 (c) in the main text. **(b)** and **(c)** The individual contributions of the two terms of equation (S2) are plotted for all illumination times. The capacitive charging contribution (B1, B2) is shown in (b) and the faradaic charging contribution (C1, C2) in (c). **(d)**, **(e)** and **(f)** Values for the fitting parameters. At the different illuination times.

In potentiometric sensing, we observe different slopes for different illumination times and the potential reaches a minimum value of approx. -0.7 V vs. Ag/AgCl. Thus, both above-described charging processes govern the signal response and equation (S2) is used for fitting. The longest illumination time (30 s) is fitted first and the fitting parameters are used as starting parameters for next smaller illumination time. The result of the fitting is shown in Figure S7 (a). The contributions of the capacitive charging term (B1, B2) and faradaic charging term (C1, C2) are shown in Figure S7 (b) and (c), respectively. When looking at the contributions of terms including the offset of A1 at different illumination times (Figure S8), it becomes evident that the B1, B2 term never reaches a potential < -0.7 V vs. Ag/AgCl, as expect for capacitive charging. Only at longer illumination times > 10 s, the C1, C2 term starts to play a significant role. The parameter A1 fits the measured photopotential when no glucose was present (see Figure S6 (d)). It is difficult to interpret the trend of parameters B1, B2 and C1, C2 (Figure S7 (e) and f) as the response of the term is always a convolution of both parameters. However, the parameters do show a trend.

Depending on the illumination time, contributions of capacitive and faradaic charging change as follows: At short times, no or not a lot of faradaic charging occurs. Thus, the charging kinetic is governed mainly by capacitive charging and analyte diffusion. The longer the illumination time, the more the sensor is charged and therefore a more significant influence of the faradaic charging to the overall diffusion process is observed. Note also, that only at times longer than 10 s, a saturation of the OCP during illumination occurs (see Figure S7 (a)), pointing towards a strong light charging of the sensor in the non-linear, faradaic charge storage regime.[11]

In summary, longer illumination times improve the sensitivity for low analyte concentrations. For measurement times between 10 s and 30 s, the slope for the range from 0 to 1 mM is much larger than from 10 to 50 mM (see Figure S7 (a)). For longer illumination times, the sensor works more accurately for low concentrations. This fits nicely with the enhanced sensitivity in this range and underlines that our sensor works best at these measurement conditions. Large glucose concentrations are measured more accurately at short illumination times below 4 s. Measurement and suitable fitting ranges are summarized in Table S1.

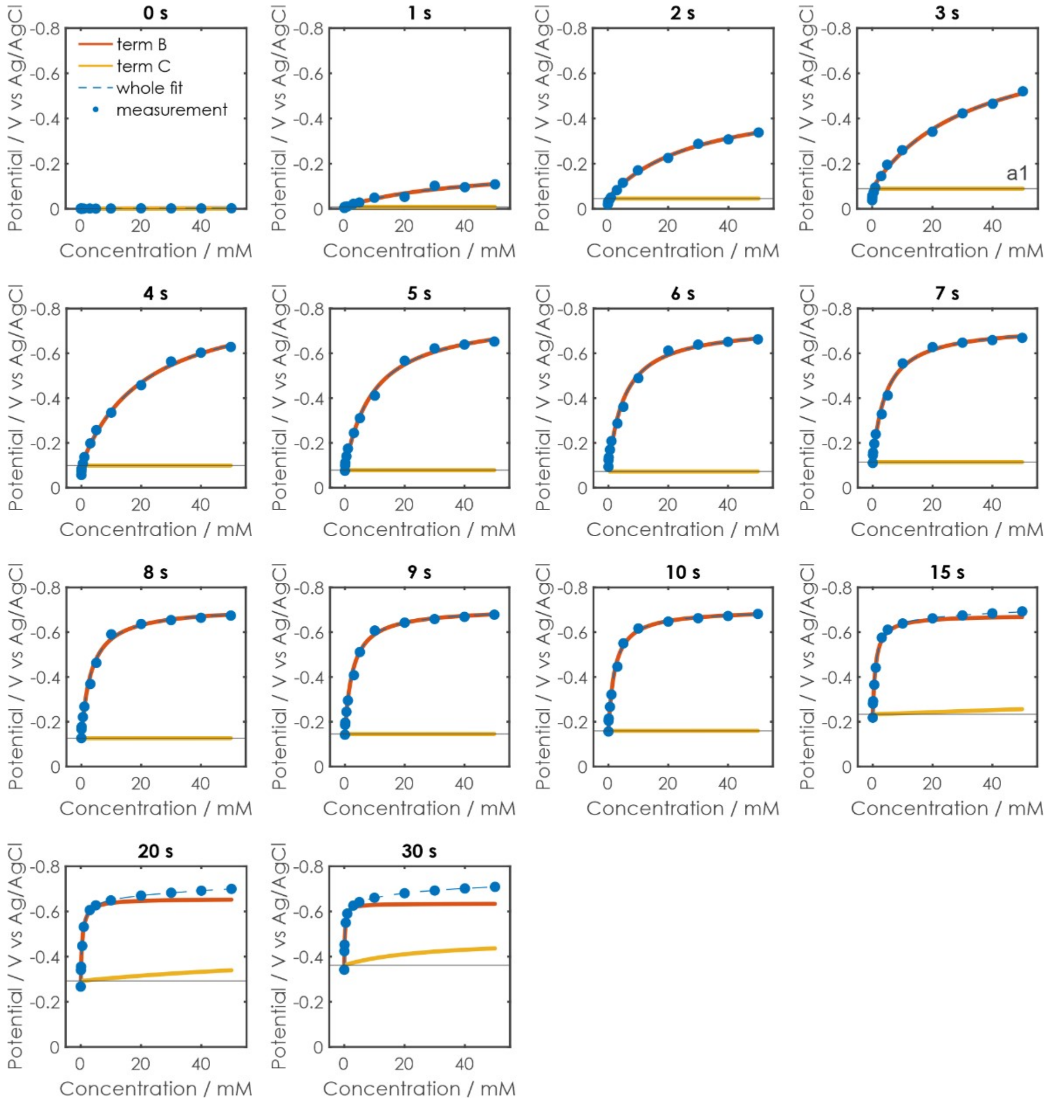

Figure S8. Individual contributions to non-linear fitting of the potentiometric sensing measurement. Comparison of measurement data (blue dots) to the whole fit (blue dashed line) and contributions of terms of B1, B2 (capacitive charging, red line) and C1, C2 (faradaic charging, yellow line) at all analyzed illumination times.

Linear Fitting

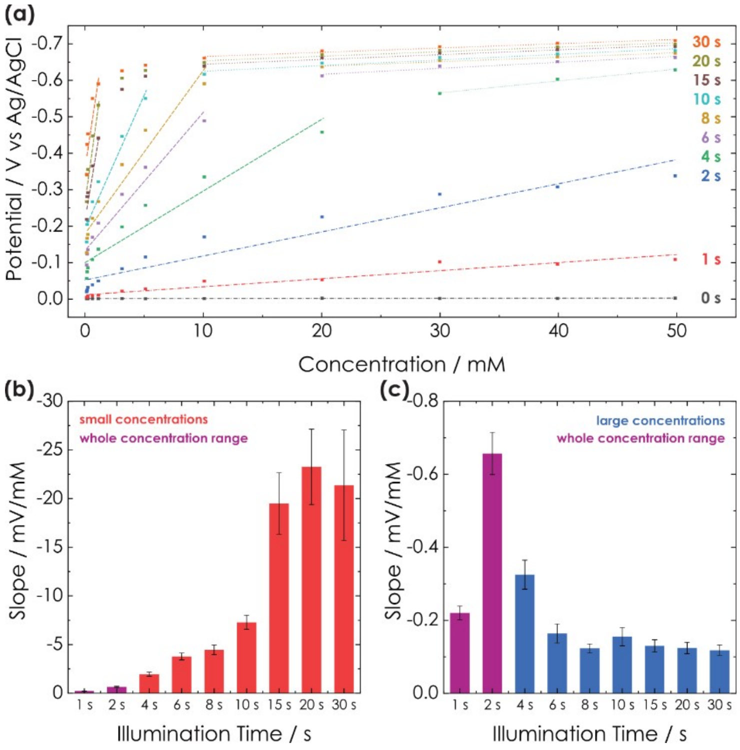

Figure S9. Linear ranges in the potentiometric sensing measurement. (a) The OCP change with glucose concentration is plotted after different illumination times. This measurement is discussed in the main text in Potentiometric sensing. Linear ranges that fulfill the fitting quality requirement of R2 > 0.9 can be found. Linear ranges for small concentrations are plotted with dash lines, linear ranges for large concentrations with dot lines and linear ranges over the entire concentration range with dash-dot lines. (b) Slopes of linear fits for small concentrations at different illumination times. (c) Slopes of linear fits for large concentrations at different illumination times. Errors in (b) and (c) have been calculated from the linear regression.

Here, we look at potentiometric sensing in which the OCP change can be linked to the glucose concentration (see main text discussion Potentiometric sensing). Depending on the illumination time, different slopes can be observed in different glucose concentration regimes. We have fitted linear regressions to different suitable concentration ranges. The respective ranges were chosen so that the fitting error of the linear regression (R2) was > 0.9. Results of this analysis are shown in Figure S9 and Table S2. When looking at the OCP increase with concentration (Figure S9 (a) and Figure 3 (c)), we can observe a roughly linear increase of the OCP over the entire measured concentration range for the first 2 s. For longer illumination times, a saturation of the OCP at large concentrations occurs at potentials < -0.7 V vs Ag/AgCl, which corresponds to the charge storage potential of K-PHI.[11] A second linear range develops with a far smaller slope. Thus, the first linear range can be attributed to the surface capacitive contribution of the OCP up to the charge storage potential of K-PHI and the second linear range to the faradaic charging of K-PHI, which causes a constantly smaller rise of the OCP. At potentials more negative

than -0.7 V vs Ag/AgCl, faradaic charging becomes the dominant kinetic process compared to the capacitive charging.

As explained in the main text, longer illumination times improve the sensitivity for low analyte concentrations. For measurement times between 10 s and 30 s, the slope for the linear range from 0 to 1 mM is much larger than from 10 to 50 mM (compare Figure S9 (b) and (c)). This means that for longer illumination times, the sensor works more accurate for low concentrations. This fits nicely with the enhanced sensitivity in this range discussed in the main text. On the other hand, large glucose concentrations are measured more accurately at short illumination times < 4 s, were the slope of the linear range for large concentrations from 10 to 50 mM is larger. This is also in line with the upper sensitivity limit discussed in the main text and allows us to define the optimal measurement settings for our sensor via potentiometric readout, which is given in Table S1.

Note, that especially all whole and small concentration ranges do not show a nice linear fit, since the system does not really behave in a linear fashion. Non-linear fitting, which is discussed above, makes much more sense for potentiometric sensing and works much better in regard to the fitting error. It also works for all illumination times over the entire concentration range.

Table S2. Linear ranges of potentiometric sensing. The linear range and fitting error R2 of the linear ranges, which were fitted to the OCP change with concentration shown in Figure S9.

**Illumination Time**	**linear range**		**R^2**
1 s	**whole range**	0.05 mM to 50 mM	0.93
2 s	**whole range**	0.05 mM to 50 mM	0.93
4 s	**small concentrations**	0.05 mM to 20 mM	0.93
	**large concentrations**	30 mM to 50 mM	0.99
6 s	**small concentrations**	0.05 mM to 10 mM	0.95
	**large concentrations**	20 mM to 50 mM	0.95
8 s	**small concentrations**	0.05 mM to 10 mM	0.93
	**large concentrations**	20 mM to 50 mM	0.98
10 s	**small concentrations**	0.05 mM to 5 mM	0.95
	**large concentrations**	10 mM to 50 mM	0.93
15 s	**small concentrations**	0.05 mM to 1 mM	0.93
	**large concentrations**	10 mM to 50 mM	0.95
20 s	**small concentrations**	0.05 mM to 1 mM	0.92
	**large concentrations**	10 mM to 50 mM	0.96
30 s	**small concentrations**	0.05 mM to 1 mM	0.83
	**large concentrations**	10 mM to 50 mM	0.96

5.2. Impedimetric sensor

Non-linear fitting

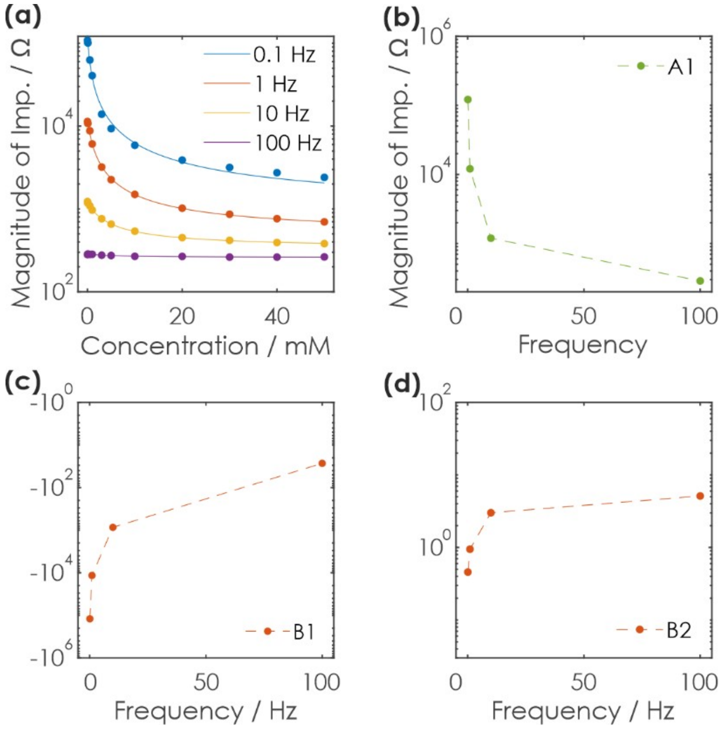

Figure S10. Non-linear fitting of the impedimetric sensing measurement. **(a)** Fitting of the magnitude of impedance was performed according to equation (S1). The fits possess an error R2 of 0.993, 0.997, 0.992 and 0.943 for frequencies of 0.1, 1, 10 and 100 Hz, respectively. Illumination time is fixed to 30 s. **(b)**, **(c)** and **(d)** Fitting coefficients of the fit.

Fitting of the impedimetric readout works different to the other readout methods, since not only the charging governs the readout but also the frequency at which the sensor readout is probed. When performing sensing via impedance readout, all samples were illuminated for 30 s (see main text, impedance sensor fitting). When looking at the potential reached after 30 s of illumination (Figure S7 (a)), except of the very small concentration value it is always < -0.7 V vs Ag/AgCl. Hence, all processes which govern the kinetics of the system participate. However, the processes have different time constants in impedance which complicates the fitting. We found that equation (S1) is sufficient to phenomenologically describe the behavior reasonably well with R^2 values > 0.99 for frequencies < 10^2 Hz. The fitting and fit coefficients are shown in Figure S10.

At low frequencies < 10^1 Hz, we have observed a much steeper slope for small concentrations < 1 mM. Measurements in this range can thus be performed with a better accuracy. The good fit quality for this frequency range (R^2 = 0.99) allows the most reliable readout of the sensing information. The loss in

sensitivity at faster frequencies, discussed in the main text, is also accompanied by a loss in measurement accuracy. This suggests that impedance measurements at frequencies > 10^2 are less accurate for determination of the analyte concentration. In fact, the change in magnitude of impedance is much smaller than the measurement error over the entire concentration range already for 100 Hz and measurements should be performed < 10 Hz. To summarize, with slower frequencies a better sensitivity and accuracy is achieved for both slow and large concentration. However, large concentrations can not be measured well for any frequency due to the very small slope. This result is represented in Table S1.

Linear fitting

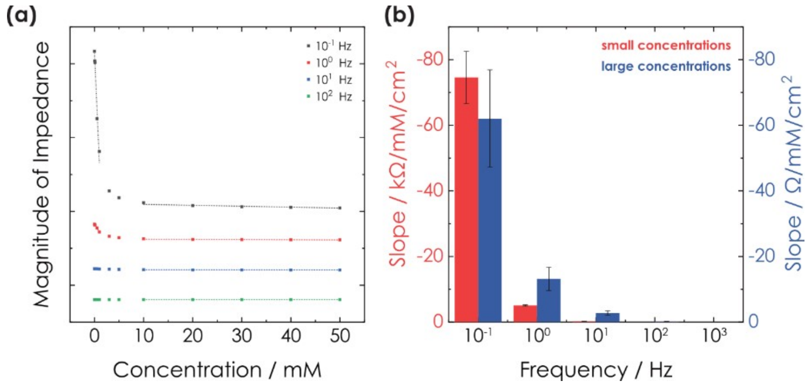

Figure S11. Linear Ranges in Impedance sensing. **(a)** The magnitude of the impedance was measured for different glucose concentrations and discussed in the main text Impedimetric sensing. Two linear regressions for two different concentration ranges were performed: 0 to 1 mM (dash-dot lines) and 10 to 50 mM (dash lines). **(b)** Slope extracted from the linear regressions of the concentration ranges, plotted for every frequency. Errors were calculated from the linear regression.

The impedance sensing measurements that are discussed in the main text in Impedance sensing were fitted with two linear ranges. The fitting and respective slopes are shown in Figure S11 and fitting ranges as well as fitting errors are given in Table S3. At low frequencies < 10^1 Hz, we have observed a much steeper slope for small concentrations < 1 mM than for large concentrations. Measurements in this range can thus be performed with a better accuracy. The loss in sensitivity at faster frequencies discussed in the main text is also accompanied by a loss in measurement accuracy. The very small R^2 values for those measurements (red numbers in Table S3) are caused by the very small slope and thus, those frequencies are not useful to extract the sensing information accurately. The slopes for large concentrations > 10 mM show a similar behavior of larger slopes for slower frequencies. Note, that they are three orders of magnitude smaller and thus much less accurate than for small concentrations. To summarize, with slower frequencies a better sensitivity and accuracy is achieved for both slow and large concentrations. However, the fitting quality especially for larger frequencies is still much worse than non-linear fitting which is discussed above and should be used instead for readout. Note, that non linear fitting also works over the entire concentration range. These results are summarized in Table S1.

Table S3. Linear ranges of impedance sensing. The linear range and fitting error R2 of the linear ranges, which were fitted to the impedance change with concentration shown in Figure S11.

Frequency	linear range		R^2
10^{-1} Hz	small concentrations	0.05 mM to 1 mM	0.97

	large concentrations	10 mM to 50 mM	0.85
10^0 Hz	small concentrations	0.05 mM to 1 mM	0.99
	large concentrations	10 mM to 50 mM	0.82
10^1 Hz	small concentrations	0.05 mM to 1 mM	0.99
	large concentrations	10 mM to 50 mM	0.84
10^2 Hz	small concentrations	0.05 mM to 1 mM	0.04
	large concentrations	10 mM to 50 mM	0.61
10^3 Hz	small concentrations	0.05 mM to 1 mM	0.54
	large concentrations	10 mM to 50 mM	0.68

5.3. Coulometric sensor

Non-linear fitting

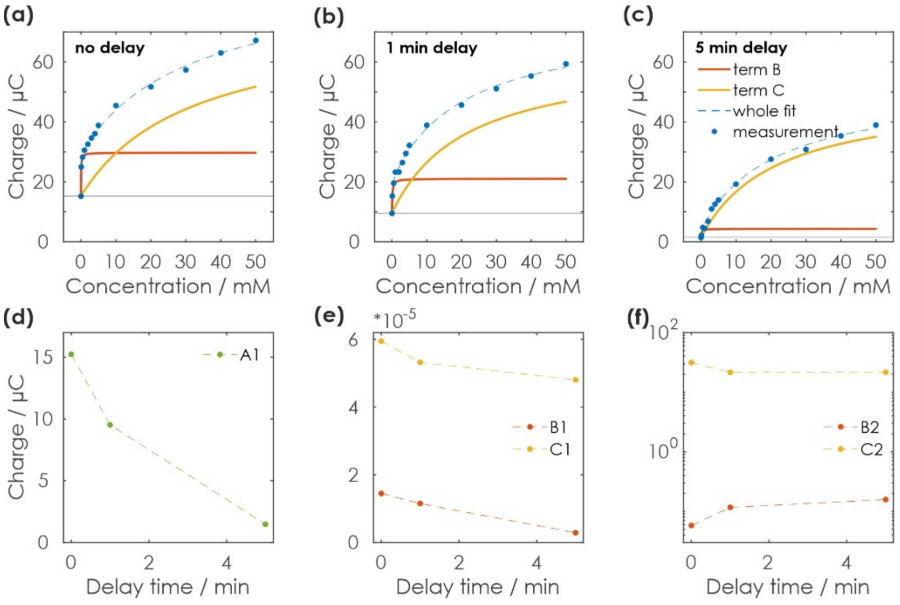

Figure S12. Non-linear fitting of the coulometric sensing measurement. **(a)**, **(b)** and **(c)** Fitting of the extracted charge after the sensing interaction, i.e., coulometric sensing was performed according to equation (S2). The fits possess an R2 of 0.997, 0.996 and 0.997 for delay times of 0, 1 and 5 min, respectively. **(d)**, **(e)** and **(f)** Fitting coefficients of the fits at different delay times.

For coulometric sensing, the sensor was illuminated for 120 s. From potentiometric sensing discussed in section 5.1, we know that after already 30 s the potential reached values < -0.7 V vs. Ag/AgCl for nearly all analyte concentrations and both capacitive as well as faradic charging contributed to the charging process. Both processes should thus also influence charging in the coulometric measurements and we have therefore, used equation (S2) to fit the sensor response. Note that for longer illumination times, the charge should increase even more.

The fit for different delay times is shown in Figure S12 (a)-(c). The shape of the two fitting terms b and c looks similar to the potentiometric fitting shown in Figure S8. With longer delay times, mainly term b decreases due to the slow self discharge discussed in the main text. For small analyte concentrations there

is nearly no charge left after the delay of 300 s, term b gets very small. Term c, which contributes to the faradaic capacity, does not decrease that much or change a lot in shape. Thus, we can calculate a parameter to correct for delayed discharging from the decrease of term b. Fitting parameters are given in Figure S12 (d)-(f). The very good fit quality ($R^2 > 0.996$) hints that a reliable and accurate readout of the sensing information is possible throughout the entire measured analyte concentration range. A summary of fitting results is given in Table S1.

Linear fitting

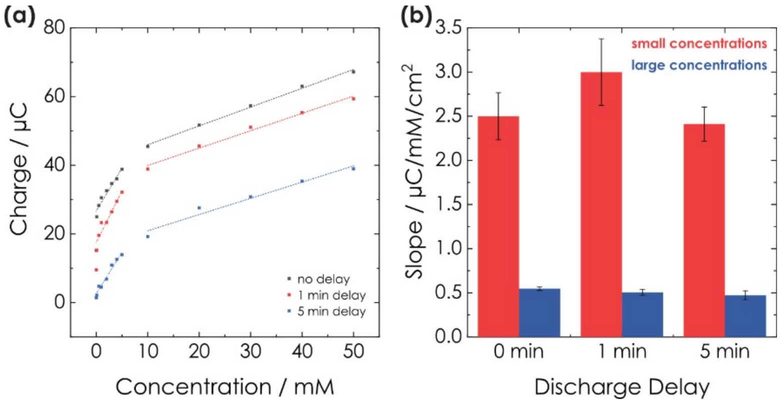

Figure S13. Linear Ranges in Coulometric sensing. **(a)** The extracted charge after illumination was measured for different glucose concentrations and discussed in the main text Coulometric sensing. Two linear regressions for two different concentration ranges were performed: 0.1 to 5 mM (dash-dot-dot lines) and 10 to 50 mM (dash lines). **(b)** Slope extracted from the linear regressions of the two concentration ranges, plotted for every frequency. Errors were calculated from the linear regression.

The link between extracted charge after charging the sensor and glucose concentration is discussed in the main text Coulometric sensing. The results of fitting linear ranges to this data and their slopes are shown in Figure S13. Concentration ranges as well as the fitting error R^2 are given in Table S4. Two linear ranges were attributed, one for small concentrations (0.1 to 5 mM) and one for large concentrations (10 to 50 mM). The slope for smaller concentrations is steeper, which gives it a better sensing accuracy. An advantage of this method in comparison to potentiometric and impedimetric sensing is the larger linear concentration range up to 5 mM compared to 1 mM for small concentrations. Similar to potentiometric and impedimetric sensing, non-linear fitting again works much better than linear fitting in regard to the fitting error and also works over the entire concentration range. The best sensing conditions are given in Table S1.

Table S4. Linear ranges of coulometric sensing. The linear range and fitting error R2 of the linear ranges, which were fitted to the charge change with concentration shown in Figure S13.

**Delayed readout**	**linear range**		**R^2**
0 s	small concentrations	0.1 mM to 5 mM	0.95
	large concentrations	10 mM to 50 mM	1.00
60 s	small concentrations	0.1 mM to 5 mM	0.93
	large concentrations	10 mM to 50 mM	0.99
300 s	small concentrations	0.1 mM to 5 mM	0.97

	large concentrations	10 mM to 50 mM	0.97

The delay times of 60 and 300 s do not alter these slopes significantly. Mean and standard deviation of the linear fits of both concentration ranges calculated from all delay times are given in Table S5. This means, that memory sensing does not influence accuracy of the linear fitting significantly.

Table S5. Slopes of linear fits. The mean and standard deviation calculated from the linear fits of 0, 60 and 300 s delayed discharge.

Value	small conc. (0.1 mM to 5 mM) [µC mM^{-1}]	large conc. (10 mM to 50 mM) [µC mM^{-1}]
mean	2.83	0.509
standard deviation	0.288	0.0377

5.4. Colorimetric sensor

Non-linear fitting

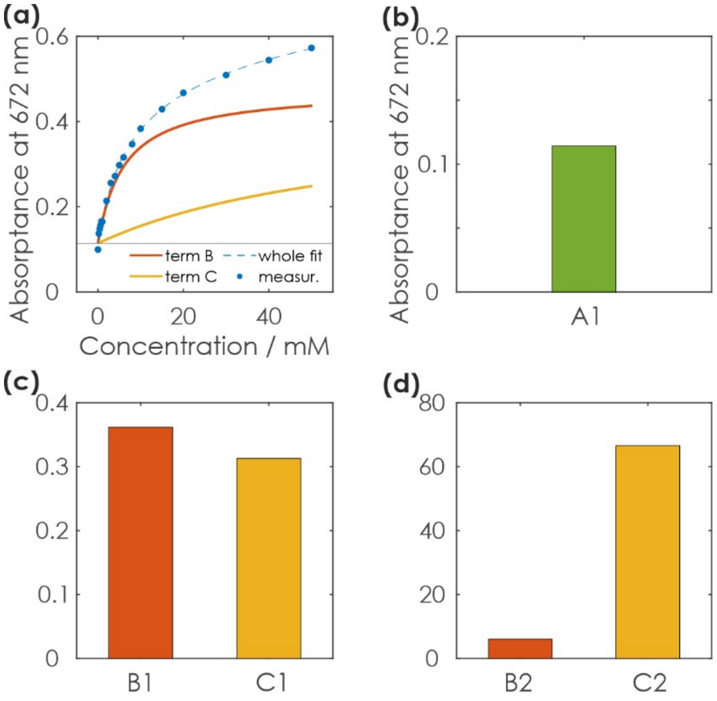

Figure S14. Non-linear fitting of the colorimetric sensing measurement. **(a)** Contributions of term b and c of equation S2 to the fitting of the absorptance signal at a wavelength of 672 nm after illumination. The fit possesses a fitting error R2 of 0.999. **(b)**, **(c)** and **(d)** Corresponding fit coefficients.

Fitting of the optical sensor readout methods works analogous to fitting of the electrochemical potentiometric and coulometric sensing with equation (S2), as the charging mechanism of the wireless particles is similar. Since the sample was illuminated for 200 s, both capacitive and faradaic charge storage mechanisms contribute to the charging (see Figure S8: For illumination times of 30 s, nearly all

concentration measurements reach a potential < -0.7 V vs. Ag/AgCl), in agreement with the potentiometric fitting. The fitting is shown in Figure S14. The shape of the two terms B1, B2 and C1, C2 looks similar to the potentiometric sensor fitting for long illumination times (Figure S8). Term b relates to the capacitive charging and term c to the faradaic charging. Fit coefficients are shown in Figure S14 (b)-(d). The very good fit quality (R^2 of 0.999) allows a reliable readout of the analyte concentration over the entire measured concentration range. Best sensing conditions are summarized in Table S1.

Linear fitting

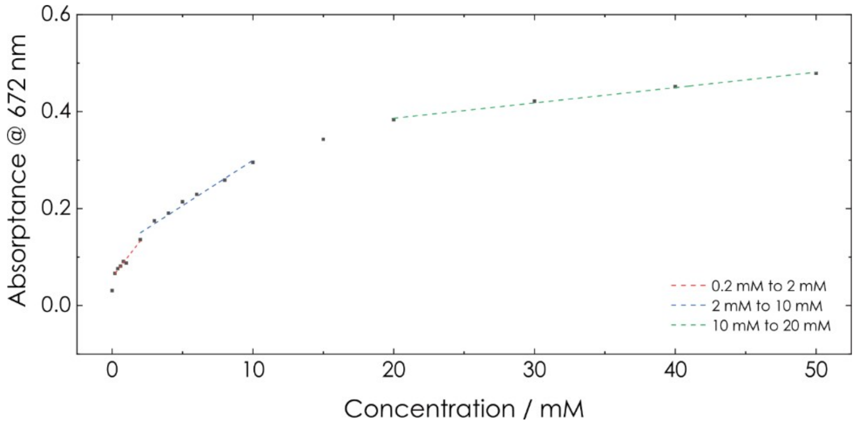

Figure S15. Linear Ranges in colorimetric sensing. The absorptance signal at a wavelength of 672 nm after illumination was measured for different glucose concentrations, as described in the main text Colorimetric sensing. Three linear regressions for three different concentration ranges were performed.

Analogous to fitting linear ranges to electrochemical sensor readout methods described above, we have fitted three linear ranges to concentration ranges of the absorptance signal, which was measured of a K-PHI suspension after charging and is described in the main text Colorimetric sensing. Ranges were chosen so that the fit quality criterion ($R^2 > 0.9$) is fulfilled. The fits are shown in Figure S15. Three different concentration ranges can be observed, slopes are given in Table S6. The slope of the fit decreases with increasing analyte concentration. Therefore, with larger analyte concentrations the sensing accuracy decreases. Note, that non-linear fitting discussed above works better than linear fitting in regard to the fitting error and also works over the entire concentration range. Best sensing conditions are summarized in Table S1.

Table S6. Fitting parameters for linear ranges of coulometric sensing. The linear range and fitting error R2 of the linear ranges, which were fitted to the absorptance change at a wavelength of 672 nm with concentration shown in Figure S15.

Linear range	Slope [Absorptance mM^{-1}]	R^2
0.2 mM to 2 mM	0.037 ± 0.003	0.97
2 mM to 10 mM	0.019 ± 0.001	0.98
20 mM to 50 mM	0.003 ± 0.000	0.99

5.5. Fluorometric sensor

Non-linear fitting

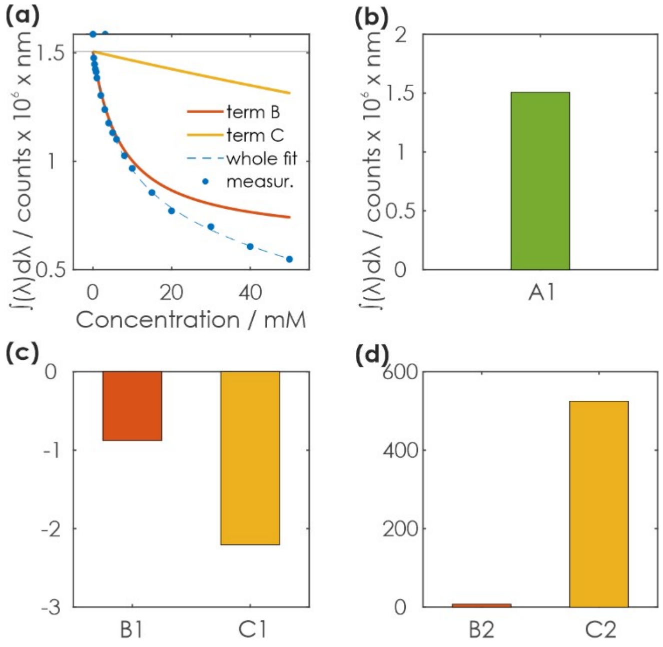

Figure S16. Non-linear fitting of the fluorometric sensing measurement. **(a)** Contributions of term b and c of equation S2 to the fitting of the absorptance signal at a wavelength of 672 nm after illumination. The fit possesses a fitting error R2 of 0.998. **(b)**, **(c)** and **(d)** Corresponding fitting coefficients.

The fitting of the fluorometric sensor readout works analogous to the colorimetric sensor discussed in section 5.4 via equation (S2). The terms b and c of the fitting are shown in Figure S16 (a) and show a similar behavior to the colorimetric readout (Figure S14 (a)) and potentiometric as well as coulometric readout (Figure S8 and Figure S12 (a)). Both capacitive and faradaic charge storage plays a role since the illumination time during the charging is 200 s, as discussed for the colorimetric sensor in section 5.4. Fitting coefficients are shown in Figure S16 (b)-(d). Due to the very good fitting quality (R^2 = 0.998), a reliable concentration readout throughout the entire measured analyte concentration range is possible. In Table S1, the best sensing conditions are summarized.

Linear fitting

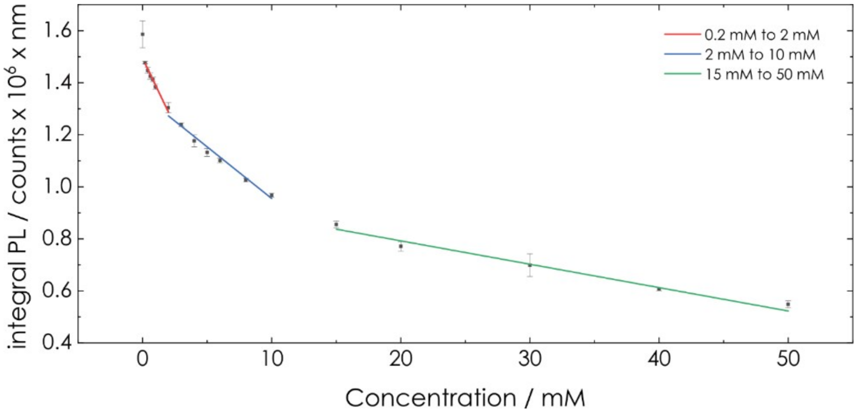

Figure S17. Linear Ranges in fluorometric sensing. The PL signal after illumination was measured for different glucose concentrations and integrated, as described in the main text Fluorometric sensing. Three linear regressions for three different concentration ranges were performed.

Analogous to the linear fitting of the colorimetric sensor discussed above, we have fitted three linear ranges to concentration ranges of the integrated photoluminescence (PL) signal, which was measured of a K-PHI suspension after charging and is described in the main text Fluorometric sensing. Ranges were chosen so that the fit quality criterion ($R^2 > 0.9$) is fulfilled. The fits are shown in Figure S17. Three different concentration ranges can be observed and their slopes are given in Table S7. With larger analyte concentrations, the slopes get smaller and thus, the sensing accuracy decreases. Similar to colorimetric sensing, the non-linear fitting works again better than the linear fitting in regards to the fitting error. It also works over the entire concentration range and should therefore be used. Sensing conditions are summarized in Table S1.

Table S7. Fitting parameters for linear ranges of fluorometric sensing. The linear range and fitting error R2 of the linear ranges, which were fitted to the integrated PL signal change with concentration shown in Figure S17.

Linear range	Slope [counts x 10^6 mM^{-1}]	R^2
0.2 mM to 2 mM	-0.104 ± 0.006	0.99
2 mM to 10 mM	-0.040 ± 0.002	0.99
15 mM to 50 mM	-0.009 ± 0.0007	0.98

6. Additional electrochemical analysis of memristive properties of K-PHI films

6.1. Delayed coulometric sensor information readout

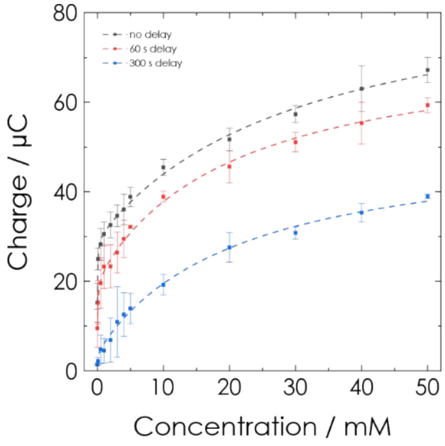

Figure S18. Analysis of delay times for memory sensing. Charge extracted from the sensor via coulometric readout after a delay of 0, 60 and 300 s (black, red and blue points, respectively). The dashed lines show the phenomenological non linear fit discussed in SI section 5.3. The measurement with no delay is also shown in the main text in Figure 3 (g).

In the main text *Coulometric sensing*, a time delayed readout of memristive properties is discussed for coulometric readout. After charging the sensor during sensing, the readout via discharging is delayed by 60 and 300 s. Results are shown in Figure S18. A decrease in extracted charge for all analyzed concentrations can be observed, which we explain with self discharge via water reduction or oxygen leaking into the reactor (see main text *Coulometric sensing* for more details). This decrease produces a systematic offset for fitting (see SI Section 5.3) and a correction parameter becomes necessary to still allow a readout. The signal decay was calculated via the measurement at a concentration of 50 mM. Results are shown in Table S8.

Table S8. Impact of delayed readout on signal intensity. Coulometric sensing charge signal loss after the delayed readout compared to the measurement with no delay. Measurement shown in Figure S18.

Delay time [s]	Signal loss in charge [%]
60	11.7
300	42.0

7. Additional optical analysis of memristive properties of K-PHI particles

7.1. Influence of particle size

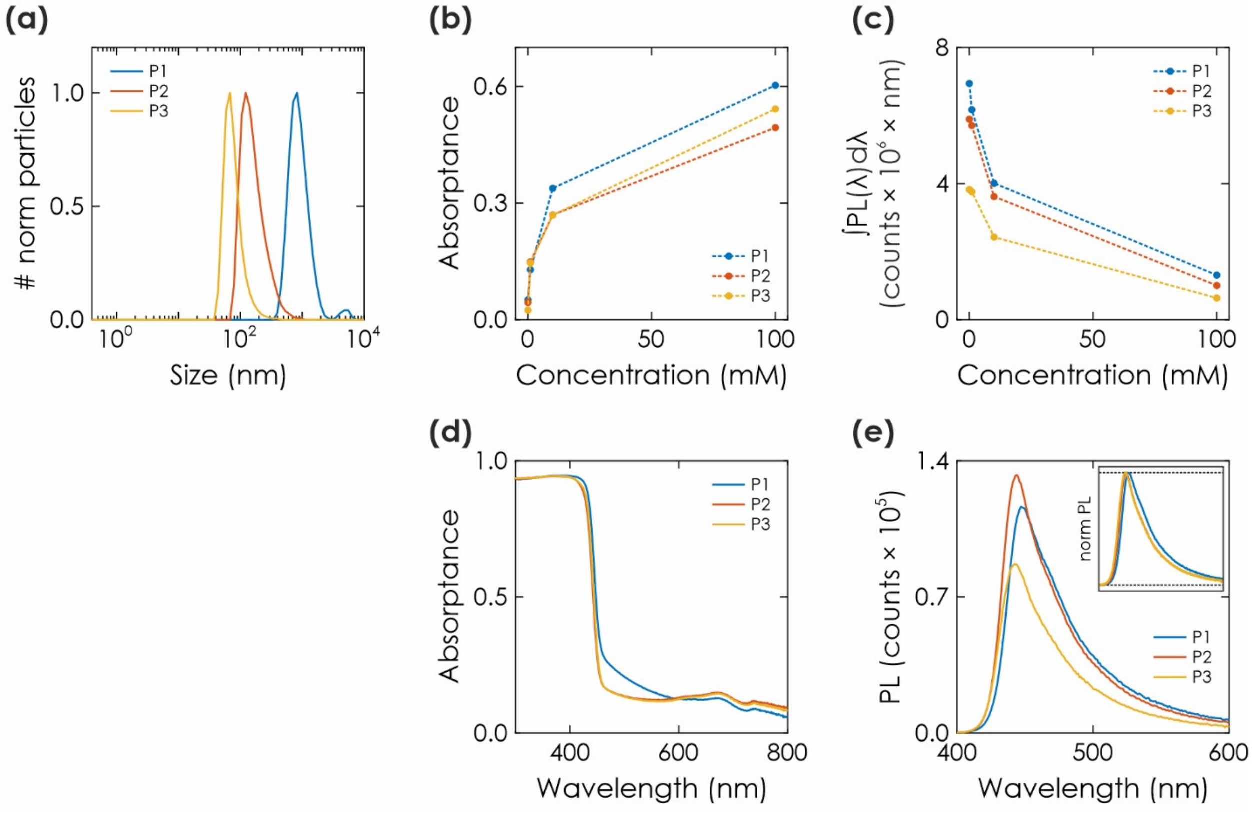

Figure S19. Influence of particle morphology on sensing efficiency. (a) Particle size distributions after centrifugation of fractions P1, P2, and P3, achieved by exfoliating as-synthesized and washed K-PHI via sonication for 2 h in an ice bath and subsequent exfoliation at 2,000 rpm for 25 min, 3,000 rpm for 40 min and 24,000 rpm for 90 min, respectively. Sensing is performed under analogous conditions as measurements shown in the main text (Figure 4 and 5), but at a lower particle concentration of 2 mg/mL. **(b)** Output of colorimetric sensing comparing the suspensions containing particles P1, P2, and P3 via change in absorptance at a wavelength of 672 nm (compare to main text Figure 4 (c)). **(c)** Output of fluorometric sensing with particles P1, P2, and P3 via integrated PL emission signal (compare to main text Figure 5 (b)) **(d)** Absorptance of particle suspensions P1, P2, and P3 before the sensing experiment. (e) PL emission of particle suspensions P1, P2, and P3 (excitation at 370 nm) before the sensing experiment (inset show the PL spectra normalized to P1).

To evaluate influence of particle size on the sensing response, we perform sensing experiments with particle suspensions, which have different particle sizes. As described in the Methods section in the main

text, we use different centrifugation steps to separate the nanosheets from the bulk material. Here, we use the different particle fractions from the individual centrifugation steps to yield particles sizes in the range of 1200 nm (P1), 250 nm (P2), and 150 nm (P3) – as reported in our previous work.[11,14] Dynamic light scattering measurements (DLS) of the different particle sizes are shown in Figure S19 (a). We performed colorimetric and fluorometric sensing measurements in analogous conditions to the measurements presented in Figure 4 and 5 in the main text (glucose concentrations of 1, 10, 100 mM), but with a slightly smaller concentration of the K-PHI suspension of 2 mg/mL vs. 3 mg/mL, since it is difficult to obtain a very dense P3 suspension. Results are shown in Figure S19 (b) and (c).

The largest particles P1 show slightly increased absorptance and integrated emission signals (Figure S19 (a) and (b)). For the colorimetric measurement, P3 shows a larger absorptance than P2 for large concentrations of 100 mM (Figure S19 (b)). In comparison, the lowest PL integrated emission signal results from P3 (Figure S19 (c)). To conclude, P1 works best for colorimetric sensing via absorptance, but at the same time shows the smallest sensing response for fluorometric sensing since the integrated emission is the largest (Figure S19 (c)). Thus, colorimetric sensing and fluorometric sensing seem to show opposite trends. To elucidate on the cause of this behaviour, we show absorptance and emission spectra of the K-PHI suspension prior to the measurements (Figure S19 (d) and (e)). While absorptance at 672 nm remains mainly unaffected by different particle sizes, the emission shows major differences in intensity (intensity: P2 > P1 >> P3). We explain this deviation with differences in the optical density of suspensions: When preparing the suspensions, we ensured that they have the similar particle concentration of 2 mg/mL. While suspension density is the same, optical density affecting the overall PL quantum yield is according to Figure S19 (e) not the same. Other deviations influencing the PL emission intensity in Figure S19 (e) are also possible, such as partial particle sedimentation for large P1 particles or a different scattering behavior occurring during the measurement. Thus, colorimetric readout seems to be the more precise method to gauge particle size effects, suggesting that P1 particles actually give the largest sensing signal. This aligns with observations of influence of particle size effects on photocatalytic measurements.[14] Note, that since these particles are too large to deposit onto substrates, we chose the smallest particles for the photoelectrochemical sensor measurements presented herein.

Overall, the trends described in the manuscript remain unaffected by particle size, and can be regarded as small fluctuations, which do not play a role after calibration of a given sensor batch. Thus, this underlines that other particle sizes can be used with same trends, but slightly different absolute signal intensities, since suspension stability, scattering, and intrinsic photophysical properties enabling charge accumulation are not fundamentally different when modifying the particle size.[14]

7.2. Dependence of illumination time

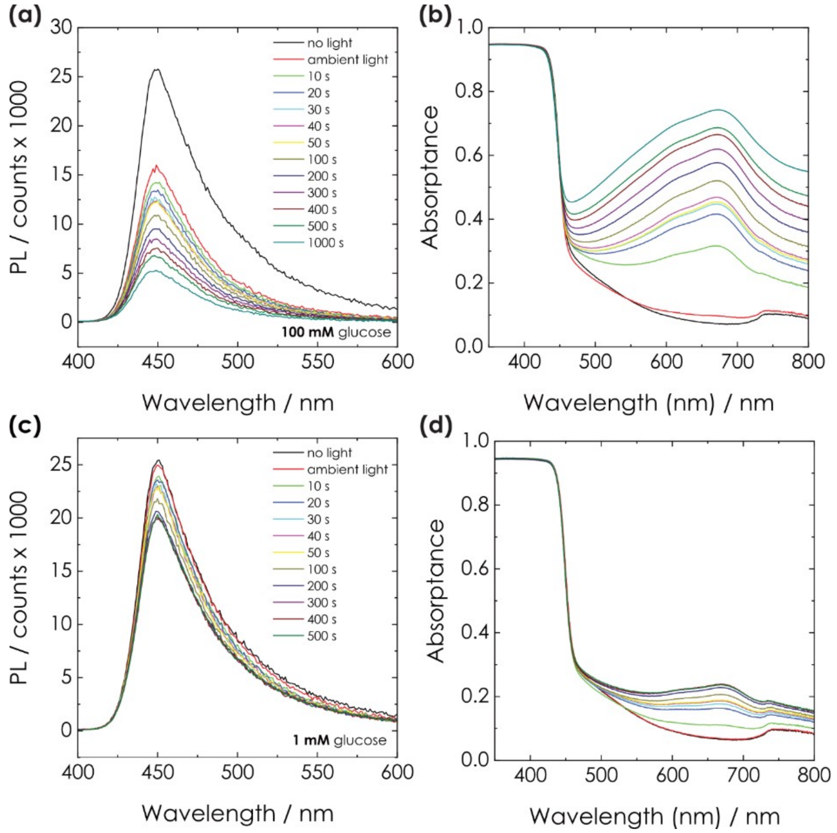

Figure S20. Evolution of optical properties with increasing the illumination time. **(a)** and **(b)** PL emission (a) and absorptance (b) after charging the K-PHI autonomous sensing particles in presence of 100 mM glucose with different illumination times. **(c)** and **(d)** PL emission (c) and absorptance (d) of an autonomous, not wired K-PHI sensor (K-PHI particles in an aqueous suspension) in presence of 1 mM glucose after charging it by different illumination times.

As discussed in the main text in *Designing a universal oxidative and memristive sensing platform*, the amount of charging of the K-PHI sensor depends on both, the glucose concentration and illumination time. In Figure S21, this illumination time is varied from 0 to 1000 s for two different glucose concentrations (1 and 100 mM). With increasing illumination time, the emission is decreasing and absorptance is increasing. Thus, the sensitivity can be tuned by varying the illumination time. For the main text measurements, a

charging time of 200 s was chosen because it represents a good compromise between signal intensity and measurement time.

7.3. Delayed sensor information readout

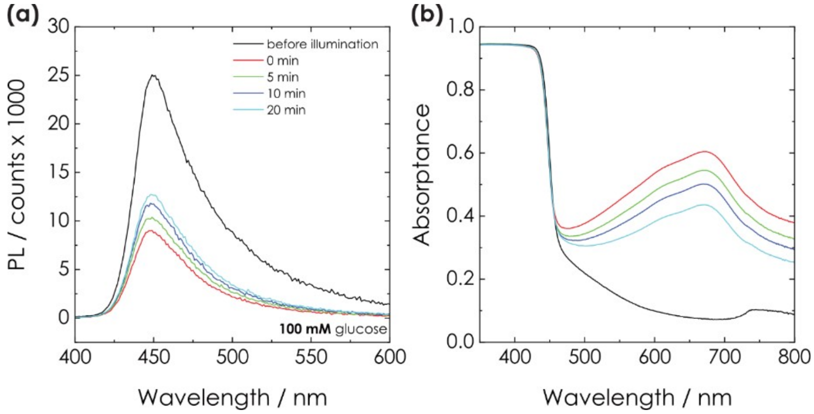

Figure S21. Analysis of delay times for memory sensing. **(a)** Emission and **(b)** absorptance of a K-PHI sensor after illuminating it for 200 s and waiting for several different delay times.

In SI section 6.1, a time delayed readout of memristive properties is discussed for coulometric readout. Here, we show that this is also possible for the optic measurement methods. A K-PHI suspension which contained 100 mM was measured under similar conditions as discussed in the main text *Memristive sensing with optical readout*. However, the optic readout after illumination was delayed by 5, 10 and 20 minutes. The measurement is shown in Figure S22. While the signal decays with longer illumination times, a readout of the optic properties is still measurable. Note that for 5 min delay, the signal loss is much less than in the wired case (42.0 %). The signal decay is given in Table S1. A delayed readout is therefore well possible if the decay is corrected. We attribute the signal decay to oxygen slowly leaking into the cuvette and quenching the excited state of K-PHI. The water reduction reaction can be excluded as mechanism here, since energy storage on K-PHI is stable at potentials negative of the RHE[11] and is only mediated by contact to conductive substrates like FTO. However, other electron accepting reactants that affect the electron storage on K-PHI and act as electron acceptors, such as oxygen in particular, should be avoided for efficient and long term charge accumulation.[3,11,15]

Table S9. Impact of delayed readout on signal intensity. Optic sensing property signal loss after the delayed readout compared to the measurement with no delay. Measurement shown in Figure S22.

Delay time [min]	Signal loss in emission [%]	Signal loss in absorptance [%]

5	8.1	11.2
10	15.5	19.4
20	21.1	31.8

7.4. Negligible invasive nature of the optical memristive state readout

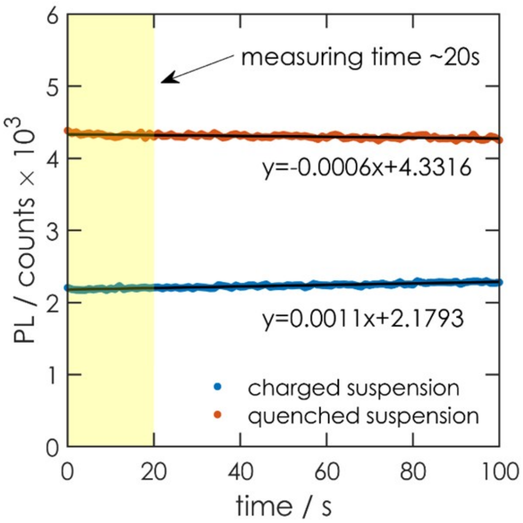

Figure S22. Kinetic photoluminescence study. Photoluminescence emission intensity (λ = 450 nm) under continuous excitation (λ = 370 nm) for the quenched with air (orange) and charged (blue) suspensions. Linear fittings, together with their corresponding equations, are shown as solid black lines and the equations given in the graph. The yellow rectangle indicates the time required to collect each PL spectrum shown in Figure 4 (b).

To proof the negligible invasiveness of the optical characterization shown in the main text, a kinetic (continuous) photoluminescence experiment was performed for 100 s to study the influence of the excitation source on the photocharging properties. This experiment was carried out under the same conditions as the PL measurements. Figure S19 show the photoluminescence remains almost constant for the quenched with air (orange curve) and for the charged (blue curve) suspensions under these illumination conditions for much longer times (100 s) than that necessary for the characterization itself (20 s). Note, that it is not necessary to measure the entire emission spectrum to extract the sensing information, since the readout of the memristive state can also be extracted at a single wavelength. Thus, the measurement time can be reduced to below 1 s (e.g., when measuring at 450 ± 2 nm), further minimizing the little disturbance of the system by its PL analysis.

In the absorption experiments case, no invasiveness is expected since the measurement is carried out by sweeping the excitation monochromator from low to high energies, hence beginning below the band gap of K-PHI. The charged state of the sensor would be modified as soon as it is illuminated with light with an energy above the bandgap (for K-PHI: λ < 450 nm), which is after the sensor information is read out (λ = 672 nm).

7.5. Schematic representation of the PL characterization process by the quenched PL emission

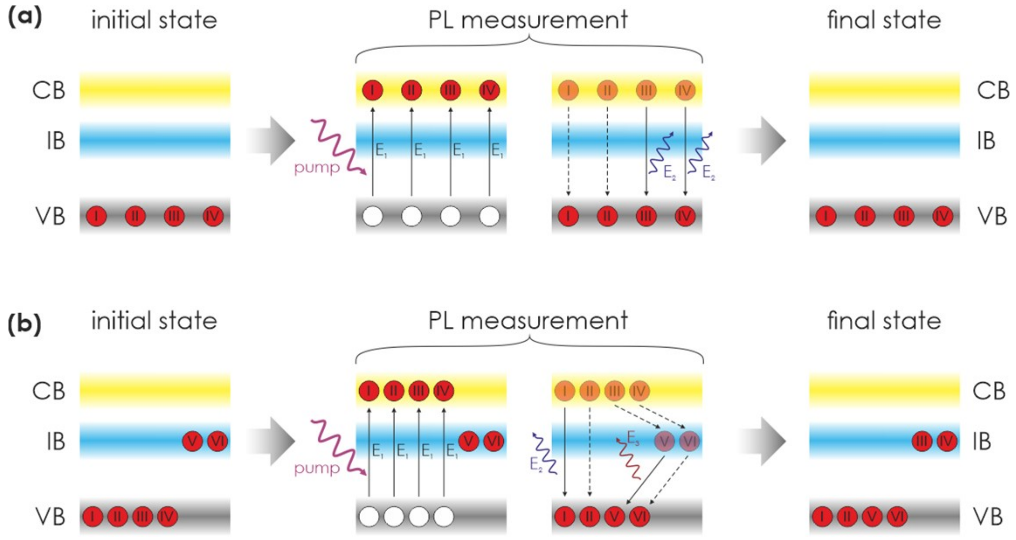

Figure S23. Proposed PL decay mechanism and its potential modification due to photocharging. Schematic representation of the PL characterization of with oxygen quenched **(a)** and charged **(b)** K-PHI suspensions in presence of glucose. Red circles represent electrons, white circles holes and the roman numbers help to visualize the path of excited electrons. The valence band (VB), conduction band (CB) and intra band (IB) are shown in gray, yellow and blue, respectively.

As mentioned in the main text, we attribute the PL intensity decline in the charged state to an either radiative at frequencies out of our detection limits (> 900 nm) or non-radiative recombination rate between photogenerated charge carriers (i.e. the holes) required for the PL measurement and increased amounts of previously trapped electrons, that increase the recombination probability. Figure S20 summarizes the measurement process for the quenched (Figure S20 (a)) and the charged (Figure S20 (b)) suspensions.

When illuminating the suspension during the PL experiment which was quenched with oxygen after a sensor experiment (Figure S20 (a)), an excited electron originating from the PL measurement (I, II, III and IV) can decay to the VB with an energy E_2 either in a non-radiative (I and II) or in a radiative way (III and

IV). The presence of oxygen in the suspension prevents the decay of these electrons to the IB. Therefore, the initial and final states of the sample under study must be similar.

In the 'charged' samples case (Figure S20 (b)), i.e., with electrons trapped in the IB (V and VI), a new recombination pathway opens between them and holes created by the PL measurement pumping (white circles). This process with energy E_3 is either non-radiative (VI) or emits beyond our detection limit (V, >900nm). Thus, less electrons relax from CB into VB, the emission with energy E_2 declines. An increased charge recombination of photoexcited electrons in charged carbon nitride photocatalysts due to much faster recombination kinetics (~400-fold) was also reported by J. R. Durrant *et al.*, in line with this proposed mechanism.[16]

8. References

1 H. Schlomberg, J. Kröger, G. Savasci, M. W. Terban, S. Bette, I. Moudrakovski, V. Duppel, F. Podjaski, R. Siegel, J. Senker, R. E. Dinnebier, C. Ochsenfeld and B. V Lotsch, *Chem. Mater.*, 2019, **31**, 7478–7486.

2 V. W. H. Lau, D. Klose, H. Kasap, F. Podjaski, M. C. Pignie, E. Reisner, G. Jeschke and B. V Lotsch, *Angew. Chemie Int. Ed.*, 2017, **56**, 510–514.

3 V. Sridhar, F. Podjaski, J. Kröger, A. Jiménez-Solano, B.-W. Park, B. V. Lotsch and M. Sitti, *Proc. Natl. Acad. Sci.*, 2020, **117**, 202007362.

4 S. J. Sadeghi, in *Encyclopedia of Biophysics*, Springer, Berlin Heidelberg, Berlin, Heidelberg, 2013, pp. 61–67.

5 G. Gauglitz, *Anal. Bioanal. Chem.*, 2018, **410**, 5–13.

6 V. W. H. Lau, I. Moudrakovski, T. Botari, S. Weinberger, M. B. Mesch, V. Duppel, J. Senker, V. Blum and B. V Lotsch, *Nat. Commun.*, 2016, **7**, 10.

7 X. Wang, K. Maeda, A. Thomas, K. Takanabe, G. Xin, J. M. Carlsson, K. Domen and M. Antonietti, *Nat. Mater.*, 2009, **8**, 76–80.

8 T. Banerjee, F. Podjaski, J. Kröger, B. P. Biswal and B. V. Lotsch, *Nat. Rev. Mater.*, 2021, **6**, 168–190.

9 H. A. Krebs, *Annu. Rev. Biochem.*, 1950, **19**, 409–430.

10 W. Göpel, T. A. Jones, M. Kleitz, J. Lundstrom and T. Seiyama, *in Ullmann's Encyclopedia of Industrial Chemistry,* Wiley-VCH Verlag GmbH & Co. KGaA, Weinheim, 2016, Chemical and Biochemical Sensors, 1. Fundamentals.

11 F. Podjaski, J. Kroger and B. V Lotsch, *Adv. Mater.*, 2018, **30**, 1705477.

12 T. Islam and S. C. Mukhopadhyay, *Int. J. Smart Sens. Intell. Syst.*, 2019, **12**, 1.

13 L. De Vico, M. H. Sørensen, L. Iversen, D. M. Rogers, B. S. Sørensen, M. Brandbyge, J. Nygård, K. L. Martinez and J. H. Jensen, *Nanoscale*, 2011, **3**, 706–717.

14 J. Kröger, A. Jiménez-Solano, G. Savasci, V. W. Lau, V. Duppel, I. Moudrakovski, K. Küster, T. Scholz, A. Gouder, M. L. Schreiter, F. Podjaski, C. Ochsenfeld and B. Lotsch, *Adv. Funct. Mater.*, **2021**, 31, 2102468.

15 V. Sridhar, F. Podjaski, Y. Alapan, J. Kröger, L. Grunenberg, V. Kishore, B. V. Lotsch and M. Sitti, *Sci. Robot.*, **2022**, 7, eabm1421.

16 W. Yang, R. Godin, H. Kasap, B. Moss, Y. Dong, S. A. J. Hillman, L. Steier, E. Reisner and J. R. Durrant, *J. Am. Chem. Soc.*, 2019, **141**, 11219–11229.
